# Exploring individual biases in BCI research and users: Does gender matter?

**DOI:** 10.3389/fnhum.2025.1695370

**Published:** 2026-04-17

**Authors:** Cornelia Herbert, Viviana Ramos Acuna, Raphael R. K. Kneipp, Nina I. Kapfer

**Affiliations:** Applied Emotion and Motivation Psychology, Institute of Psychology and Education, Ulm University, Ulm, Germany

**Keywords:** Brain-Computer Interfaces, gender bias, sex differences, electroencephalography, literature review, women in science, career perceptions, STEAM

## Abstract

**Objective:**

Brain-Computer Interface (BCI) is an interdisciplinary research field characterized by rapid technological advances and collaborative efforts to develop user-friendly, adaptive devices that enable healthy and non-responsive users to communicate and interact with their environment through brain signals elicited by specific instructions or tasks. However, research often shows gender bias, especially in scientific disciplines with strong technological, medical, or social foundations. Gender biases have been found among scientists conducting and publishing research. They may also exist among examiners and study participants.

**Research question and methods:**

This study investigates whether gender biases are present in BCI research, particularly in the distribution of women and men across editorial boards and authorship of studies focusing on psychological human factors that influence BCI performance and usability. We systematically analyzed the gender distribution in neuroscientific journals that accept BCI research or have a strong focus on BCI, reviewed their editorial boards, analyzed BCI publications –including those related to psychological human factors–and examined gender biases among study participants. Additionally, we reviewed EEG studies investigating sex- or gender-related differences in EEG signals relevant to BCI research.

**Results:**

We observed significant differences in the representation of women and men among editorial board members and BCI authors, including first-, co-, and last-authorship. Similarly, there were differences in the gender distribution of participants in BCI studies. Moreover, the literature review suggests potential differences in brain signals between women and men within the studied samples. The impact of these differences on performance in BCIs, such as motor-imagery SMR-BCIs, SSVEP-BCIs, and P300-BCIs, as well as training methods and BCI usability, still needs to be explored.

**Conclusion:**

Our findings emphasize the importance of increasing awareness of gender-, sex-, and user-related factors in BCI research. In line with recent perspectives that highlight the need to address gender biases and individual differences in the language of the user, their motivation or cultural background, future BCI research should focus on systematically examining gender and sex differences. This will help promote gender equality in BCI research and lead to a better understanding of users’ needs, preferences, and individual characteristics.

## 1 Introduction

Brain-Computer Interface (BCI) is an interdisciplinary field of research characterized by rapid technological advances and collaborative efforts to develop user-friendly devices that enable people to communicate and interact with their environment via brain signals. In most BCI settings, BCI users perform specific cognitive or emotional tasks. Performance in these tasks is required to allow automatic classification of brain signals evoked during these tasks. Brain signals from the users are recorded, for example, using non-invasive EEG devices, and converted into computer commands (Edlinger et al., 2015; Nicolas-Alonso and Gomez-Gil, 2012). Originally developed to enhance communication for non-responsive patients, BCI applications have been continually expanded to mental health care, workplace support, education, learning, and the personal lives of healthy users (Kawala-Sterniuk et al., 2021; Trott et al., 2025).

### 1.1 Gender bias in scientific research

Research has uncovered gender biases in academic research and within academia itself (Cameron and Stinson, 2019; Card et al., 2023; Casad et al., 2021; Gruber et al., 2021; Holman et al., 2018; Hopkins-Doyle et al., 2024; Larivière et al., 2013; Llorens et al., 2021; Odic and Wojcik, 2020). Currently, about 30% of researchers worldwide are women. Disciplines with a strong STEM (Science, Technology, Engineering, and Math) foundation are especially affected by gender biases. However, gender disparities also exist in scientific domains such as medicine, social sciences, and the arts (STEM or STEAM), where women remain underrepresented in senior roles despite outnumbering male students. This trend also applies to disciplines like psychology, where, as in medicine, women constitute the majority of students, earn more doctoral degrees, but may still hold less research prominence than their colleagues (Cameron and Stinson, 2019; Gruber et al., 2021; Hopkins-Doyle et al., 2024; Odic and Wojcik, 2020). Gender biases in science are not just statistical anomalies; they tend to reflect broader norms rather than being exceptions. Gender biases are significant, socially and culturally constructed stereotypes about gender that start early in education and academic careers (Cameron and Stinson, 2019; Casad et al., 2021; Gruber et al., 2021; Holman et al., 2018; Reuben et al., 2014). Due to their prevalence, stereotypes can influence the preferences, attitudes, beliefs, and decisions of children, parents, teachers, students, academic administrators, and scientists at all levels of their careers. They can cause individuals to self-select into specific scientific fields, avoiding or approaching engineering, physics, and mathematics.

Gender driven selections and gender stereotypes, but also the lack of female role models, may support the impression of many people that science and technology are mainly male domains. Both women and men often conform to gender stereotypes, which can affect their career progression and success. Recent survey studies indicate that women are more likely to engage in informal academic work, such as informal mentorship (Reuben et al., 2014; Rossi, 1965). This often goes unnoticed by their male colleagues or is taken for granted by the students they supervise. These gender-related differences in attitudes, beliefs, and behavior may discourage young women from pursuing academic careers with the same motivation, self-esteem, and empowerment as their male counterparts, especially after completing their minor, major, or Ph.D., when they decide to continue to higher levels of their academic careers (Reuben et al., 2014; Rossi, 1965; Teng et al., 2025). Moreover, academia is less appealing compared to other employment options outside the academic field. Scientists in academia often earn less than their colleagues working in larger companies (Umbach, 2006). Additionally, working conditions in academia can be demanding. Academic achievement profoundly relies on funding and the pursuit of qualifications within a limited timeframe. Many early-career researchers leave academia without finishing their qualifications. Recent estimates indicate that about 30%–50% of Ph.D. students and even more postdoctoral researchers do not complete their programs (Ghundol and Muthanna, 2025; Ryan, 2025; Upchurch, 2020; Wollast et al., 2018). A significantly higher number of women than men tend to decide to leave. Among the women who remain, only about 30% hold leadership roles.

Biases between women and men are widespread among researchers and those publishing results. Reviews and meta-analyses on this topic suggest that women are less likely to be the first or last authors on papers (Araújo and Fontainha, 2017; Banks et al., 2025; Filardo et al., 2016; Fox and Paine, 2019; Helmer et al., 2017). First and last authorship is associated with prestige and leadership in research. Furthermore, studies indicate that papers authored by women may receive fewer citations than those by men, even when controlling for quality and impact (Banks et al., 2025). Additionally, some research suggests that female authors may face different standards during peer review compared to their male colleagues, who are also more frequently represented on editorial boards (Helmer et al., 2017). Furthermore, women’s achievements and contributions are often overlooked (Fox and Paine, 2019).

### 1.2 Gender bias among study participants

The unequal representation of women and men in science, along with the reasons behind it, should be taken seriously. This also applies to volunteers in scientific studies. An imbalance in the participation of women and men can impact study results and their validity. Importantly, gender biases in recruitment strategies may influence who volunteers. Evidence consistently shows volunteer biases across scientific fields like medicine, psychology, and computer science (Ayuso et al., 2019; Bennett, 1993; Henrich et al., 2010). Many health-related studies, especially those focused on mental health or chronic illnesses, tend to have more female participants (Bennett, 1993). In social sciences, volunteer recruitment often results in a predominance of female volunteers, especially in studies involving university students participating for course credit (the WEIRD population) (Henrich et al., 2010). Gender biases can be present in various scientific studies due to implicit gender stereotypes among participants that may affect their motivation and willingness to participate. For instance, women might be more inclined to volunteer for health-related research (Gokhale et al., 2015; Hackett et al., 1991). Conversely, the social stigma that technology is for men could discourage women from volunteering, particularly in such studies. This stigma might even hinder their performance. Men may feel less motivated than women to volunteer for mental health and well-being studies if they anticipate social barriers or conflicts with gender stereotypes (Gokhale et al., 2015). Differences in the performance of women and men as study participants might occur and be facilitated by the experimenter’s gender. Studies have shown gender-related experimenter biases (Chapman et al., 2018; Chhaya et al., 2023). Experimenter bias suggests that participants may respond differently depending on the experimenter’s gender. For example, participants may be more willing to reveal personal information or show emotions to an experimenter of the same gender.

### 1.3 Gender and sex differences in neuroscientific studies

Finally, gender- and sex differences have been reported in neuroscientific studies, including non-invasive electroencephalographic (EEG) studies. Many studies have found that women and men respond differently to specific tasks, exhibit distinct electrophysiological responses to emotional or social stimuli, display different patterns during motor imagery, or exhibit distinct resting-state EEG patterns–factors relevant to BCI applications (Corsi-Cabrera et al., 1993; Jausovec and Jausovec, 2010; Pan et al., 2019; Shearer et al., 1984; Subirats et al., 2018; Uvais et al., 2020). Additionally, the language and instructions used during experiments can trigger gender-biased processing, mainly if the generic masculine form is used to represent people’s genders, as is common in many gender-marked languages (Glim et al., 2023). For example, a recent study suggests that using the masculine form promotes male-biased mental representations, which in turn influence EEG recordings. The study found that employing both masculine and feminine forms can differentially influence the amplitudes of event-related brain potentials relevant for BCI classification (Glim et al., 2023).

### 1.4 Aim and scope of the present study

This study investigates gender biases in research on Brain-Computer Interfaces (BCI) and gender- and sex-related differences in EEG studies relevant to BCI research. In summary, the study will address four main objectives:

1. Examine potential gender biases in the BCI literature, focusing on neuroscientific flagship journals and BCI journals, their editorial boards, and their recent publications.
2. Examine whether gender biases in authorship are common in BCI studies that specifically focus on psychological factors and BCI performance of healthy users and patients.
3. Assess whether gender biases exist among BCI users who volunteered for those studies involving psychological factors, including research on BCI usability.
4. Explore whether the neuroscientific literature reports any gender- or sex differences in brain signals, especially EEG, and those relevant for BCIs.

## 2 Materials and methods

### 2.1 Gender in the BCI literature

#### 2.1.1 Editorial boards

An examination of gender biases in flagship neuroscientific journals that publish BCI research and in solely dedicated BCI journals provides a representative overview. Flagship journals are considered among the most influential and respected journals in the field. Their publications can influence future research directions. Moreover, the flagship journal’s editorial decisions significantly affect the prominence of the studies published. Collections such as books, special issues, research topics within a journal, and contributions to conference proceedings can further boost the scientific visibility of a research area and the author’s work. These are important for both senior and early-career researchers in networking and advancing their careers. Thus, for flagship journals and publications, we focused on those that include the words “Brain-Computer Interface,” the abbreviation “BCI,” or “Brain Machine Interface” in their title, section, or the description of the journal’s purpose and scope. Book compilations were included if they focused solely on Brain-Computer Interfaces, were clearly labeled as such, and were published within the last 5 years. We used journal finder tools to search for the flagship journals, their scope and aims, as well as for book compilations, special issues, and conferences. We used the keyword search terms “Brain-Computer Interface,” “BCI,” “Brain Machine Interface,” or combinations of these.

This initial keyword-driven search identified eleven flagship neuroscientific journals that accept BCI publications (see Table 1a). In addition, seven BCI journals with a strong focus on BCI (Table 1b), as well as one book series.^1^ The book series was published annually, highlighting the projects of the yearly BCI Award nominees presented at the international BCI conference. Only the most recent book series published in 2023 were included in the analysis of BCI journals, focusing on peer-reviewed journals (Tables 1a,b). Of the seven journals (including the book series) dedicated explicitly to BCI, four journals offered regular monthly or annual submissions. Two journals had special issues on BCI (see Table 1b). In addition, our search identified another special issue on BCI launched by the same publishing group^2^. Similar to our decision regarding book series, to avoid giving too much emphasis to a single special issue in the same journals, after reviewing the content, we decided to include only one of the two special issues from each publisher in our analysis.

**TABLE 1a:** Gender distribution among editorial board members of neuroscientific journals that include BCI research.

Journal	Editor-in-chief	Editorial board member gender (woman/man)	Percentage of women	Impact score	Cite score
Neuroimage (Elsevier)	Man/woman	22/39	36.07%	4.7	11.3
Frontiers in Neuroscience (Frontiers Media SA)	Man	229/707	24.47%	4.3	6.8
Clinical Neurophysiology (Elsevier)	Man	2/8	20.00%	3.7	8.7
Psychophysiology SPR (Wiley Online Library)	Man	13/19	40.63%	2.9	6.8
Biological Psychology (Elsevier)	Man/woman	16/35	31.37%	2.8	4.2
Journal of Neuroscience Methods (Elsevier)	Man	21/32	39.62%	2.7	7.1
Behavioral Brain Research (Elsevier)	Man/woman	16/21	43.24%	2.6	5.6
International Journal of Psychophysiology (Elsevier)	Man	13/19	40.63%	2.5	5.4
Frontiers in Human Neuroscience (Frontiers Media SA)	Man/woman	126/396	24.14%	2.4	4.7
Neuroscience and Behavioral Physiology (Springer Nature)	Man	2/9	18.18%	–	–
International Journal of Bioelectromagnetism (International Society for Bioelectromagnetism, ISBEM)	Man	1/12	7.69%	–	–

The numbers of women and men are estimated based on first names and the information provided on the journal’s homepage.

**TABLE 1b:** Gender distribution in editorial boards of BCI journals, BCI special issues, and the book series.

Journal Focus on BCI	Editor-in-chief	Editorial board member gender (woman/man)	Percentage of women	Impact score	Cite score
Brain Computer Interfaces - BCI (Taylor & Francis)	Man	2/7	22.22%	2.1	4.0
Frontiers in Human Neuroscience Section: Brain-Computer Interface (Frontiers Media SA)	Man	53/177	23.04%	2.7	5.5
Brain Informatics – Subsection BCI (Springer Open)	Man	2/35	5.40%	4.5	8.0
Nature – Subsection BCI (Springer Nature)	Woman	40/29	57.97%	50.5	–
**Journal – with a special issue on BCI**	**Editor-in-chief**	**Editorial board member gender (woman/man)**	**Percentage of women**	**Impact score**	**Cite score**
Journal of Neural Engineering Special Issue on BCI (IOP Science)	–	4/1	80%	3.8	7.8
Special Issue on Brain–Computer Interfaces: advances and challenges (MDPI, Multidisciplinary Digital Publishing Institute)	–	0/2	0%	3.5	8.2
**Book on BCI**	**Editor-in-chief**	**Editorial board member gender (woman/man)**	**Percentage of women**	**Impact score**	**Cite score**
Brain-Computer Interface Research - A State-of-the-Art Summary 12 - 2023 (Springer Briefs in Electrical and Computer Engineering)	–	1/6	14.29%	–	–

##### 2.1.1.1 Determination of the gender in the editorial boards

Next, the gender of the editorial boards in the journals was determined. The gender selection was based on the names provided and listed on each journal’s homepage. All members, including specialty chief editors, senior associate editors, associate editors, and the editorial board, were included if they were listed on the journal’s homepage (see Tables 1c,d). Review editors were not included due to their large number of members in each journal. We inferred the editors’ gender using two methods: manual checks and semi-automated preprocessing. These methods were also used in the subsequent analyses of authorship. Therefore, the detailed preprocessing steps are described in the following sections [see Section “2.1.2 BCI publications (BCI journals)” for a detailed description].

**TABLE 1c:** Overview of the types of editors for the journals and those included in the analysis (“marked with an x”).

Senior editors	x
Senior advisory boards	x
Associate editors	x
Editorial boards	x
Editor-in-chief	x
Consulting editors	x
Past-editor	
Review editors	
Statistical associate editor	x
Special issue editor	x
Field chief editors	x
Assistant field chief editors	x
Specialty chief editors	x

**TABLE 1d:** Number of editors of each BCI journal.

Journal – focus on BCI	Type	Number of editors
Brain Computer Interfaces - BCI (Taylor & Francis)	Journal	14
Frontiers in Human Neuroscience Section: Brain-Computer Interface (Frontiers Media SA)	Journal	280
Brain Informatics – Subsection BCI (Springer Open)	Subsection	53
Nature – Subsection BCI (Springer Nature)	Subsection	78
Journal of Neural Engineering Special Issue on BCI (IOP)	Special issue	6
Brain–Computer Interfaces: Advances and Challenges (MDPI, Multidisciplinary Digital Publishing Institute)	Special issue	2
Brain-Computer Interface Research - A State-of-the-Art Summary 12 - 2023 (Springer Briefs in Electrical and Computer Engineering)	Book series	8
Total		441
Final sample (after preprocessing)		359

#### 2.1.2 BCI publications (BCI journals)

To identify potential gender biases in the publications of the BCI journals (see Table 1b), we reviewed all recent articles published over the past 5 years (2019–2025). We examined authorship for the first author, co-authors, and last authors. This process involved several semi-automated data preprocessing steps. In short, preprocessing included extracting articles from regular monthly or quarterly issues, collecting the authors’ names, and noting their authorship positions (first author, coauthor, or last author). Additionally, we examined the relationships between gender and authorship positions, specifically whether publishing as a first author, coauthor, or last author was associated with different probabilities of the author’s gender across these roles. Moreover, we examined whether female last authors are more likely to publish with female first authors and female coauthors than male last authors, or if any significant relationships between authorship positions can be explained by gender. We focused on the authorship position. Often, the last author is a senior author. However, exceptions occur. Therefore, we prefer to use the term “last author” rather than “senior author”. For a detailed discussion of the advantages and disadvantages of this analysis regarding the role of senior authors, see the Sections “4 Discussion, 5 Strengths, limitations, and outlook.”

The first semi-automatic preprocessing step involved extracting Digital Object Identifiers (DOIs) from the websites of the journals for all articles. This process was implemented using the R package rvest (Wickham, 2024), which provides automated data collection from web pages. To gather metadata, such as authors’ names and publication dates, we used the Crossref API.^3^ The rcrossref package (Chamberlain et al., 2022) in R Software (version 4.3.2, R Core Team, 2023) allows automated data analysis. For the book series, author names and titles were manually added from the table of contents. To identify potentially identical author names with minor orthographic variations–such as including or omitting hyphens, diacritics, or substituting characters such as “n” for “ñ”–we calculated pairwise Jaro-Winkler (J-W) distances. The Jaro-Winkler distance measures the similarity of texts, especially names. A higher distance indicates less similarity, while a lower distance suggests greater similarity. We used the stringdist package (van der Loo, 2014) in R to compute the J-W scores. Out of a total of *N* = 4678 names extracted, 735 name pairs with a Jaro-Winkler distance below 0.15 (meaning a similarity score above 0.85) were identified and manually reviewed to confirm actual identity. For J-W scores below 0.15 (similarity scores above 0.85), we manually double-checked the names to verify their true identities (see Section “3 Results”).

##### 2.1.2.1 Determination of the gender of author names

To determine the gender associated with the authors’ names, we utilized the Gender-API^4^ and the R packages *httr* and *jsonlite* (Ooms, 2014; Wickham, 2023). The Gender-API is a comprehensive online database that links names to their respective genders, primarily using governmental data sources. The Gender-API’s definition of gender is based on the binary gender definition that views gender as a social construct that varies across societies and over time. It includes the socially constructed roles, behaviors, activities, and attributes that a given society considers appropriate for women, men, girls, and boys^5^. Thus, each name query returns a predicted binary gender (the name is more likely for a woman or a man), an accuracy score from 0 to 100, and the number of samples used for the prediction. We accepted an accuracy score of 80% (or higher) as reliable and valid to classify the results as “true/correct.”

Many scientific journals only display the author’s last name in the article header, often omitting their full first names. This limits the ability to accurately predict an author’s gender based solely on header information. Because initials are used instead of full first names and because insufficient data are available for prediction in the Gender-API, we could not determine the gender for 92 unique first names, which account for 3.19% of the total sample. As a result, for 122 authors (2.61%) across various articles in the original dataset, their names could not be assigned to a binary gender.

#### 2.1.3 Gender distribution in BCI publications on psychological human factors and their users

To examine gender biases in research on psychological human factors and their BCI users, we reviewed recent publications and reviews. We identified four primary sources (Brannigan et al., 2024; Herbert, 2024a,b; Saha et al., 2021), all published within the last 5 years (2021–2025) in leading BCI journals (see Tables 1a,b). Among these, two publications provided detailed overviews of research on psychological human factors affecting BCI performance in both healthy and vulnerable users, focusing on the most influential types of BCIs: P300, SMR, and SSVEP (Herbert, 2024a; Saha et al., 2021, see Table 2 for an overview). The third publication summarized recent BCI studies emphasizing linguistic paradigms and the language used by BCI users, including patients and healthy participants (Herbert, 2024b). The fourth publication offered a summary of BCI research focusing on user preferences and usability (Brannigan et al., 2024). All studies from these publication sources were analyzed together. In total, we found *N* = 98 studies from these four sources (Brannigan et al., 2024; Herbert, 2024a,b; Saha et al., 2021) (for an overview, see Table 2).

**TABLE 2:** Number of women and men participating in studies on psychological human factors and BCI performance.

Primary source	References	Sample size	Men sample size	Women sample size	Sample mean age	Sample age SD	Healthy sample	Non-healthy sample	Inclusion in sample analyses
Herbert, 2024a	Kleih et al., 2010	33	4	29	23.4	4.24	33		✓
Herbert, 2024a	Kleih et al., 2011	90			29.29		77	13	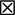
Herbert, 2024a	Kleih and Kübler, 2013	20	6	14	23.35	3.78	20		✓
Herbert, 2024a	Kleih-Dahms et al., 2021	35	15	20	24.22	3.51	35		✓
Herbert, 2024a	Nijboer et al., 2008	16	6	10	25.44	3.71	16		✓
Herbert, 2024a	Nijboer et al., 2010	7						7	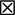
Herbert, 2024a	Baykara et al., 2016	16	8	8	23.88	2.5	16		✓
Herbert, 2024a	Ahn et al., 2018	52	26	26	24.8	3.86	52		✓
Herbert, 2024a	Hammer et al., 2012	80	39	41	29.5	11.58	83		✓
Herbert, 2024a	Hammer et al., 2014	32	18	14	24.2	2.88	33		✓
Herbert, 2024a	Hammer et al., 2018	40	21	19	25.8	8.46	40		✓
Herbert, 2024a	Jeunet et al., 2015	18	9	9	21.5	1.2	18		✓
Herbert, 2024a	Sprague et al., 2016	34					34		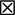
Herbert, 2024a	Leeuwis et al., 2021	57	21	36	20.71	3.52	57		✓
Herbert, 2024a	Halder et al., 2011	20	13	7	24.5	3.7	20		✓
Herbert, 2024a	Blankertz et al., 2010	80	39	41	29.9	11.5	80		✓
Herbert, 2024a	Botrel et al., 2017	154	55	99	24.7	5.8	154		✓
Herbert, 2024a	Botrel and Kübler, 2019	39					39		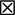
Herbert, 2024a	Jiang et al., 2021	35			38.5	15.7	35		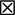
Herbert, 2024a	Bobrova et al., 2018	17					17		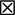
Herbert, 2024a	Müller-Putz et al., 2019								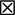
Saha et al., 2021	Romero-Laiseca et al., 2020	8	7	1			8		✓
Saha et al., 2021	Song and Kim, 2019	20	15	5	27	1.8	20		✓
Saha et al., 2021	Saha et al., 2019	5					5		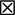
Saha et al., 2021	Toriyama et al., 2018	20	12	8			20		✓
Saha et al., 2021	Yu et al., 2018	7	6	1	26.7		7		✓
Saha et al., 2021	Darvishi et al., 2017	10					10		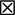
Saha et al., 2021	Park et al., 2016	12	9	3	54	6.6		12	✓
Saha et al., 2021	Kasahara et al., 2015	30	14	16	22.4	3.1	30		✓
Saha et al., 2021	Edelman et al., 2016	5	3	2	25.5	9.7	5		✓
Saha et al., 2021	Leamy et al., 2014	15	11	4	57.8	15.38	10	5	✓
Saha et al., 2021	Ramos-Murguialday et al., 2013	30	18	12	49.76	12.37		30	✓
Saha et al., 2021	LaFleur et al., 2013	5	2	3				5	✓
Saha et al., 2021	Jin et al., 2020	9	5	4			9		✓
Saha et al., 2021	Cecotti, 2020								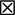
Saha et al., 2021	Halder et al., 2019	10	4	6	25.51		10		✓
Saha et al., 2021	Wei et al., 2018								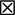
Saha et al., 2021	Guy et al., 2018	20						20	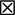
Saha et al., 2021	Alcaide-Aguirre et al., 2017	30					11	10	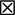
Saha et al., 2021	Waytowich et al., 2016	15	9	6	39.5		32		✓
Saha et al., 2021	Norton et al., 2015								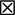
Saha et al., 2021	Botrel et al., 2015								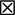
Saha et al., 2021	Farwell et al., 2014	30	15	15	26	2.9		30	✓
Saha et al., 2021	Petrov et al., 2014	12	10	2			12		✓
Saha et al., 2021	Chi et al., 2012	10							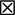
Saha et al., 2021	Iturrate et al., 2009	5					5		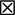
Saha et al., 2021	Oxley et al., 2021	2						2	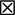
Saha et al., 2021	Oxley et al., 2017								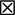
Saha et al., 2021	Oxley et al., 2016								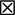
Saha et al., 2021	Sauter-Starace et al., 2019								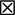
Saha et al., 2021	Mestais et al., 2015								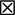
Saha et al., 2021	Sand et al., 2017								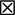
Saha et al., 2021	Kaiju et al., 2017								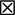
Saha et al., 2021	Vansteensel et al., 2016	1							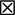
Saha et al., 2021	Downey et al., 2016	2						2	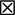
Saha et al., 2021	Pahwa et al., 2015	2	2					2	✓
Saha et al., 2021	Yin et al., 2014								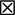
Saha et al., 2021	Keefer et al., 2008								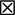
Saha et al., 2021	Friehs et al., 2006								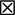
Saha et al., 2021	Zuo et al., 2020	18	12	6	23.6	3.7	18		✓
Saha et al., 2021	Sereshkeh et al., 2019								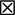
Saha et al., 2021	Corsi et al., 2019	15	8	7	28.13	4.1	15		✓
Saha et al., 2021	Chiarelli et al., 2018	15							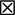
Saha et al., 2021	Ge et al., 2017	12	6	6	26		12		✓
Saha et al., 2021	Zhao et al., 2016	5	4	1	24	2.7	5		✓
Saha et al., 2021	Combaz and Van Hulle, 2015	9							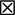
Saha et al., 2021									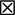
Saha et al., 2021	Grau et al., 2014	4					4		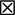
Saha et al., 2021	Choi and Jo, 2013								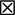
Saha et al., 2021	Kauhanen et al., 2006	3	3	0				3	✓
Herbert, 2024b	Kotchoubey et al., 2005	105						105	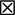
Herbert, 2024b	Schoenle and Witzke, 2005	120							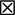
Herbert, 2024b	Steppacher et al., 2013	92							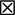
Herbert, 2024b	Rämä et al., 2010								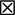
Herbert, 2024b	Balconi et al., 2013	18	10	8	50	10.11	18		✓
Herbert, 2024b	Wutzl et al., 2021								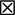
Herbert, 2024b	Aubinet et al., 2022								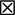
Herbert, 2024b	Chiossi and Bisiacchi, 2017								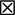
Herbert, 2024b	Herbert and Kübler, 2011	18	4	14	25.5	5.11	18		✓
Herbert, 2024b	Van Vliet et al., 2010	3	3	0			3		✓
Herbert, 2024b	Dijkstra et al., 2020								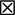
Herbert, 2024b	Wilson et al., 2023	12					12		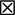
Brannigan et al., 2024	Brown-Triolo et al., 2002	94	72	22				94	✓
Brannigan et al., 2024	French et al., 2010								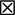
Brannigan et al., 2024	Sellers et al., 2010	1	1					1	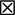
Brannigan et al., 2024	Huggins et al., 2011	61							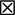
Brannigan et al., 2024	Collinger et al., 2013	57	51	6	52.66	11.01		57	✓
Brannigan et al., 2024	Zickler et al., 2013	4						4	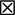
Brannigan et al., 2024	Kageyama et al., 2020	780	461	311	64.1	10.3		1918	✓
Brannigan et al., 2024	Grübler et al., 2014	19	15	4				19	✓
Brannigan et al., 2024	Huggins et al., 2015	39	27	12	52	17		39	✓
Brannigan et al., 2024	Engdahl et al., 2015	104	70	34	47	15		104	✓
Brannigan et al., 2024	Geronimo et al., 2015								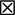
Brannigan et al., 2024	Blabe et al., 2015	156						156	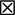
Brannigan et al., 2024	Andresen et al., 2016								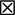
Brannigan et al., 2024	Tigra, 2019	31	25	6				31	✓
Brannigan et al., 2024	Branco et al., 2021	28						28	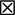
Brannigan et al., 2024	Kim et al., 2022	8	7	1				8	✓

The table offers a comprehensive overview of the sample characteristics, including the age range and whether the participants’ gender or sex was reported. The last column indicates if the information was provided  ✓; or not provided  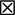.

##### 2.1.3.1 Determination of the gender of author names and BCI users

Before determining the gender of the authors and BCI users, we checked for duplicate studies. The steps for identifying the authors’ gender followed the same procedure outlined above. For analyzing the BCI volunteers in the selected studies, we manually extracted details about the BCI users. It is important to note that many studies provided insufficient information about how the volunteers’ gender was determined, such as whether gender was based on biological sex or self-identified gender. Some studies did not report any details about their volunteers. If a study did not include information about a BCI user’s self-reported gender or sex, and no additional gender data could be obtained from the article, this was counted as missing (see Section “3 Results”).

#### 2.1.4 Differences between women and men in neural processing relevant for BCI

**FIGURE 1:**
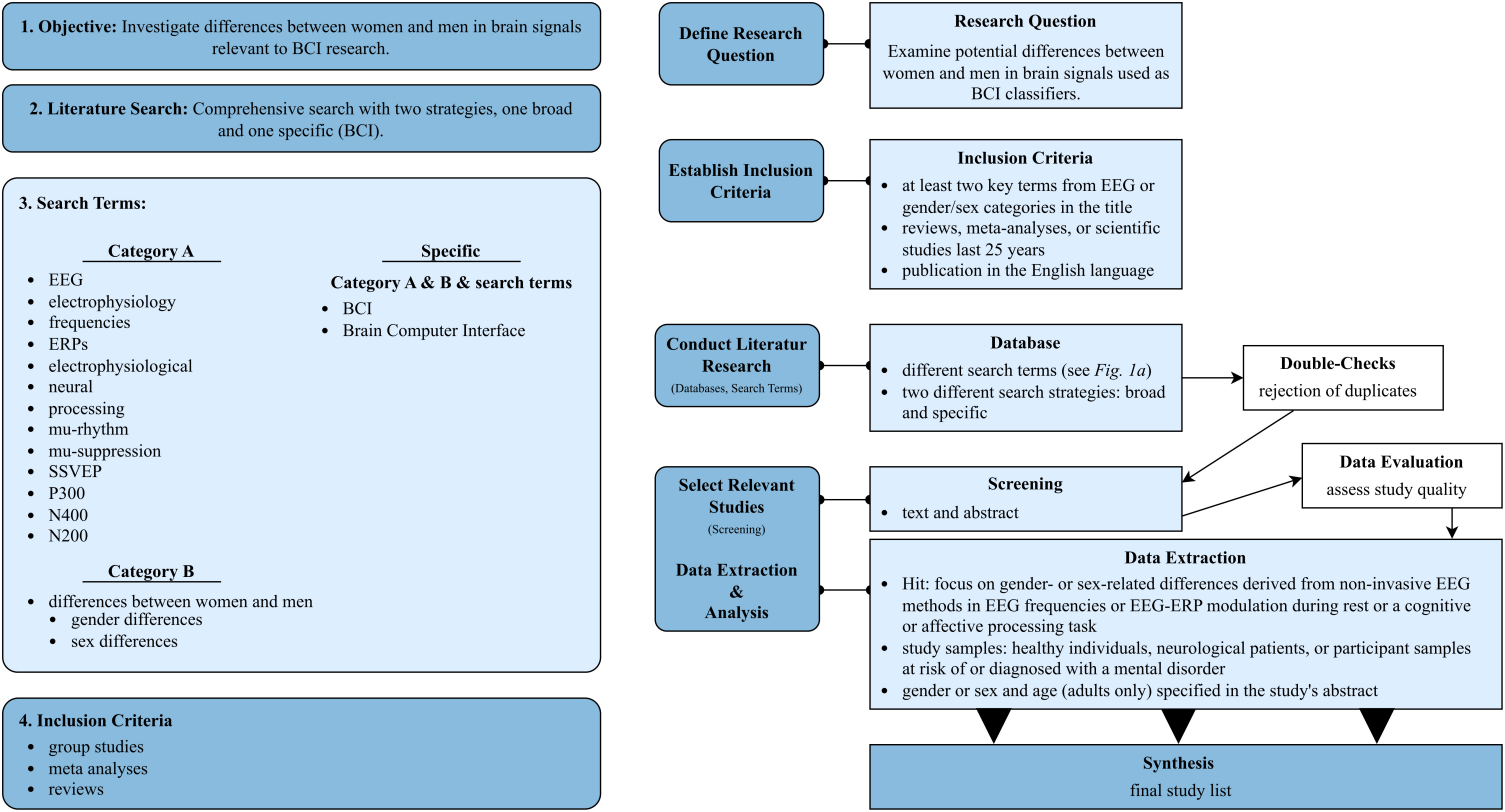
Left: Literature search of the exploratory review identifying differences in EEG brain signals between women and men. Summary of the keywords (search terms) and inclusion criteria; see the text for details. Right: Process of the literature search of the exploratory review.

A literature review was conducted to examine potential differences between women and men in brain signals relevant to EEG-based BCI classification. After a comprehensive search, we conducted a more focused search specifically on the EEG signals most frequently used in previous BCI studies involving both healthy and vulnerable users (see Figure 1 for illustration).

**FIGURE 2:**
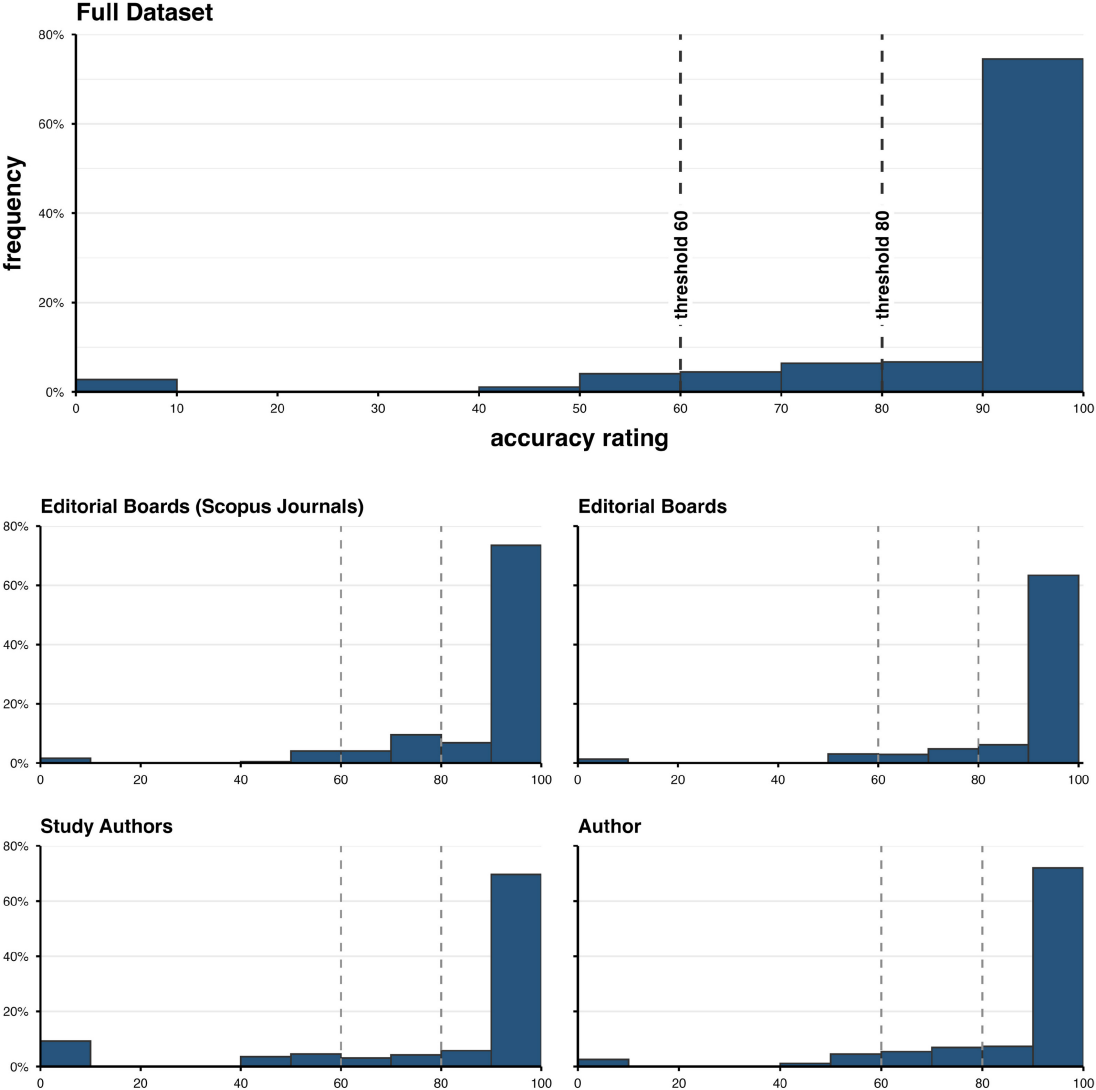
Accurate identification of gender-related first names from the semi-automatic analyses of names and the gender of editorials and authors (for details, see the text).

##### 2.1.4.1 Determination of gender- and sex-related differences

We primarily used PUBMED for the literature review. The following keywords were employed and combined as search terms: “EEG,” “frequencies,” “ERP,” “neural processing,” “electrophysiological processing,” “mu-rhythm,” “mu suppression,” “P300,” “N400,” “N200,” “differences between women and men,” “sex differences,” “gender differences,” and “BCI.” These keywords were carefully combined using “AND” or “OR” to facilitate both broad and targeted searches. Hits were counted only if at least two of the search terms (one from each category A or B) appeared in the title, and the study was published within the last 25 years (1980–2005), as shown in Figure 1. The extended time period was included to account for changes in sex- and gender-related research and publication biases. Hits from this search that included at least two of the keywords, either from EEG (A) or gender/sex categories (B) in the title, were included when they were reviews, meta-analyses, or scientific studies reporting findings from group studies (see Figure 1). Next, all hits were manually approved by screening the title and abstract. The hit remained on the list for analysis if the study, review, or meta-analysis focused on gender- or sex-related differences derived from non-invasive EEG methods in EEG frequencies or EEG-ERP modulation during rest, altered states of consciousness (e.g., sleep) or during cognitive or affective or another processing task (e.g., motor and action processing) including samples of healthy individuals, neurological patients, or participant samples at risk of or diagnosed with a mental disorder. Duplicates were rejected to prevent double-counting. The conducted literature review included studies that reported investigating gender- or sex-related differences in EEG-based neural processing. Both aspects (gender and sex) were included to explore the relevance of considering both gender- and sex-related differences in the development of BCIs and their users. To this end, we also examined whether gender and sex were defined in these studies to explore potential recommendations for BCI studies. For an overview of the procedure, see Figure 1.

In total, the keyword search combinations, with the restriction that at least two category keywords must appear in the title, yielded more than 50 hits. These were screened according to the inclusion and exclusion criteria outlined above, limiting the publications to research papers, reviews, and meta-analyses. A summary of these articles is shown in Table 11 and in detail in the Supplementary Table 1. The narrower search, which also required at least two category keywords from categories A and B (see Figure 1) to be in the title, and additionally a search term related to BCI, yielded only one hit. However, considering only one key from category A or B yielded two additional hits. After screening according to the aforementioned inclusion and exclusion criteria, these additional articles remained on the list (see Table 11 and Supplementary Table 1). We used MAXQDA^6^ to analyze the the information provided in the abstracts, preprocess hits from the literature search, and systematize reports based on keywords and results reported in the abstract. Additionally, all publications were checked both automatically and manually for the methods used to determine gender and sex, or if no information was provided. If gender or sex and age (adults only) were not specified in the study’s abstract or the manuscript (gender, sex), this information was counted as missing and as not reported (see entries “not specified” in the Supplementary Table 1. The results of this detailed review are shown in the Supplementary Table 1.

### 2.2 Statistical analysis

For all analyses described in Section “2.1 Gender in the BCI literature,” we calculated descriptive statistics for the gender distributions within the editorial boards of the leading neuroscience journals (see Table 1a) and of journals specifically focused on Brain-Computer Interfaces (BCI) (see Table 1b). For the BCI journals, we also present descriptive data and statistical analysis to evaluate potential gender biases in the authorship of the published articles. Additionally, we provide descriptive data and statistics on potential gender biases among the authors of the 98 publications discussing BCI-related psychological human factors, and report the demographic data of the BCI users in these publications, including their gender and age (for both healthy users and vulnerable users).

To assess whether the gender distribution in editorial boards, authorships, and BCI users significantly differed from a balanced ratio (i.e., 50%) or occurred by chance, we conducted binomial tests whenever gender data were available. Next, we conducted more detailed analyses to examine differences in gender distributions across journals and publications and to explore factors that might influence the representation of women and men as editors or authors. We categorized the data by several variables: for editors, we controlled for the year of editorial membership and the journal; for authors, we included the publication year (2019–2025) and authorship status (first author, coauthor, or last author). We examined whether significant gender differences exist across these variables and within the three specialized journals dedicated to BCI research that provided coherent data across all 5 years (see Table 1b).

Differences in gender distributions among editorial board members, first-, co-, and last authors, and BCI users (healthy and vulnerable users) were evaluated using various statistical tests. Chi-square tests of independence assessed whether the proportions of women and men differed across journals and publications within or among the BCI journals. Fisher’s Exact tests determined whether observed differences also applied to the first, the coauthors (middle), and the last authors. Additionally, chi-square tests and *post hoc* two-proportion *z*-tests were used to examine gender-related patterns in authorship positions. Furthermore, we classified a subset of articles based on the team composition into three categories: “same gender female,” “same gender male,” and “mixed gender.” We then explored the associations between team composition and the gender of the first and last authors using two-proportion *z*-tests. To assess whether the year of publication could predict gender, we employed logistic regression models for the three BCI journals with “publication year” as a continuous predictor. We fitted three separate models: one for the overall gender proportion among all authors, one for first authors, and one for last authors.

In all analyses, we aimed to include the complete datasets, excluding only data from names of editorial board members or authors whose gender was unknown or for names whose accuracy scores fell below the respective threshold (see Section “2 Materials and methods” for data analysis). Similarly, for some analyses, we could use only a subset of articles where the gender of all authors or BCI users was accurately determined.

The *p*-values reported for each statistical test were corrected for multiple comparisons. For correction, *p*-values were adjusted for multiple testing using the Benjamini-Hochberg correction. To avoid biasing the results toward overly conservative conclusions, we also report the uncorrected *p*-values in case the uncorrected and corrected results lead to different conclusions.

## 3 Results

### 3.1 Gender biases on editorial boards

#### 3.1.1 Neuroscientific journals

On average, a minority of the editorial board members of the eleven neuroscience journals were women. The proportion of women in these journals varied significantly, from 7.69% to 43.24%. A Fisher’s exact test of independence showed that this variation was statistically significant (*p* < 0.001). Thus, while women were underrepresented as editorial members, the degree of underrepresentation differed significantly between journals. Three journals had the highest representation, with two journals having 40.63% of women and one having 43.24% respectively. Conversely, some journals had a very low representation of women: only 7.69% of the authors were women, indicating that the total number of women in editorial boards differed significantly from an expected equal distribution of 50% (binomial test, *p* < 0.001). Regarding the role of editor-in-chief, seven out of eleven journals had a male editor in this position. Four journals listed a man and a woman as co-editors-in-chief. No journals appointed a woman solely to this role.

#### 3.1.2 BCI journals

**FIGURE 3:**
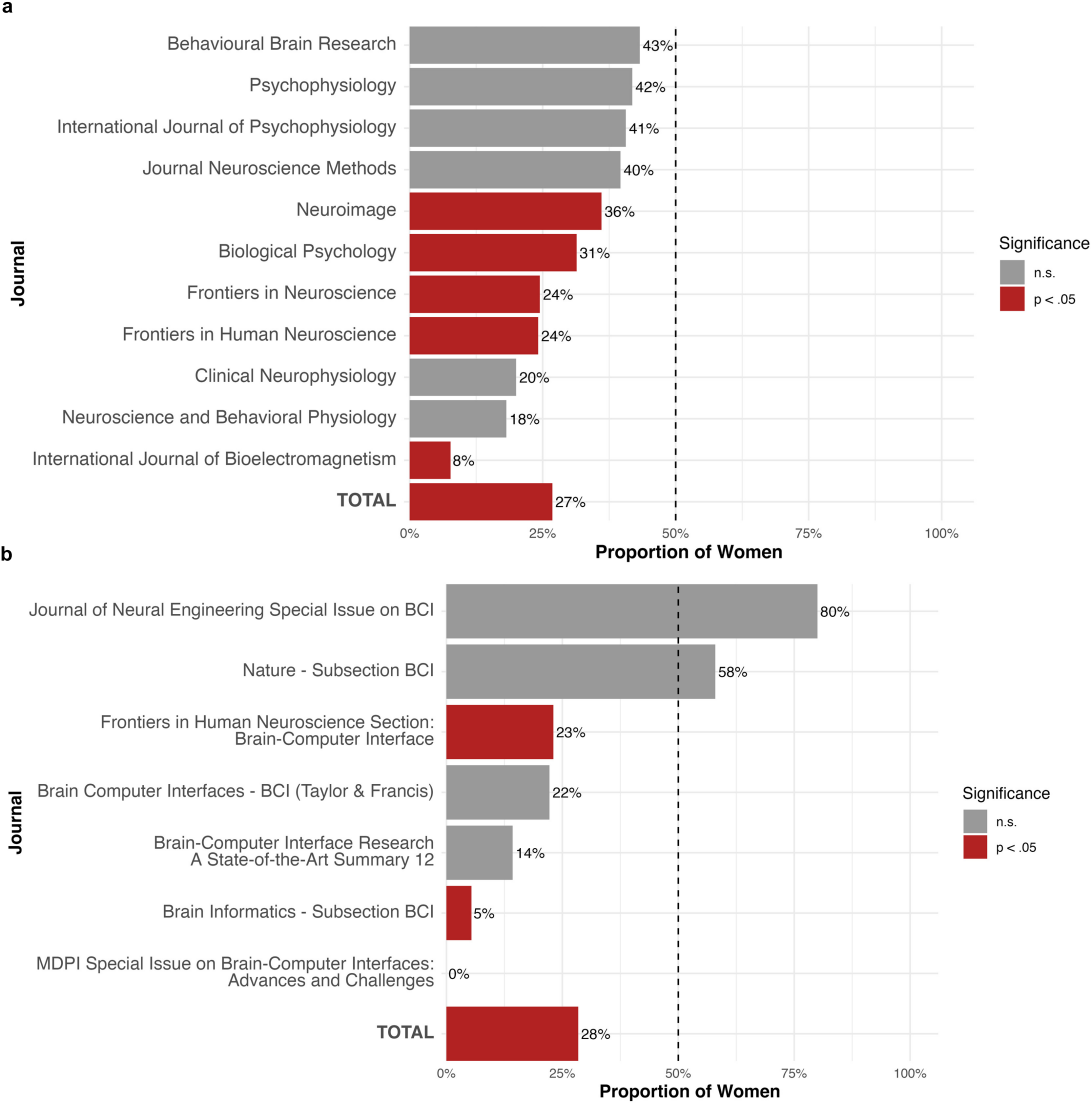
**(a)** Percentage of women on the editorial boards of the neuroscience journals. The dashed line indicates gender parity (50%). **(b)** Percentage of women on the editorial boards of the BCI journals. The dashed line indicates gender parity (50%).

For the members of the editorial boards of the journals specific to BCI (Table 1b), our analysis identified *N* = 441 names (see Table 1c). Of these, *N* = 399 could be recognized as unique first names using the Gender-API tool. For seven names (1.75%), gender could not be determined. This resulted in 1.59% of editors lacking gender information in the full dataset (see Figure 2). The final sample comprised names from *N* = 359 editors. Descriptive and statistical analyses of the gender distribution showed that 28% of the participants were women (see Figure 3b versus Figure 3a). Comparing the BCI journals, special issues, and book series revealed significant differences in binary gender representation, indicating that women are underrepresented in BCI editorial roles, including those in journals, special issues, and book series. One journal and one special issue stood out, with a higher proportion of women than men in editorial roles (see Table 1d). This included a subsection on BCI with an 80% representation of women, as well as a special issue on BCI, where four women and one man served as guest editors. The full sample of editors comprised *N* = 102 women and *N* = 257 men. This distribution differed significantly from an equal distribution (binomial test, *p* < 0.001). A Fisher’s exact test revealed significant differences in this proportion between journals (see Figure 3b). Regarding the role of the editor-in-chief, all BCI journals and all BCI special issues had a male editor in this position, and some reported having a man and a woman as co-editors-in-chief. No journals appointed a woman solely to this role.

### 3.2 Gender biases in BCI publications published in the BCI journals

**FIGURE 4:**
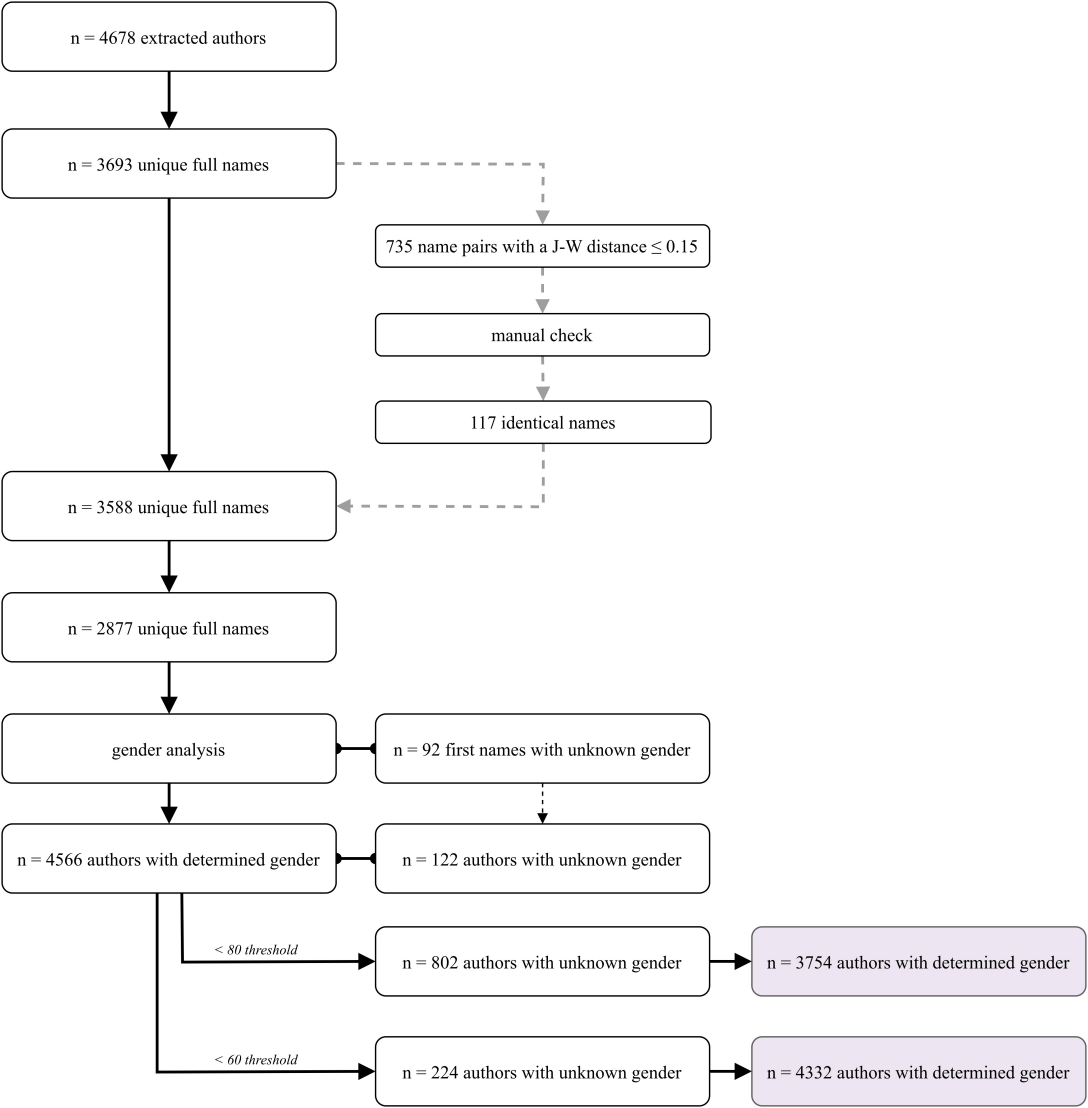
Illustration of the preprocessing steps used to determine the number of authors and their gender (for details, please see the text).

**FIGURE 5:**
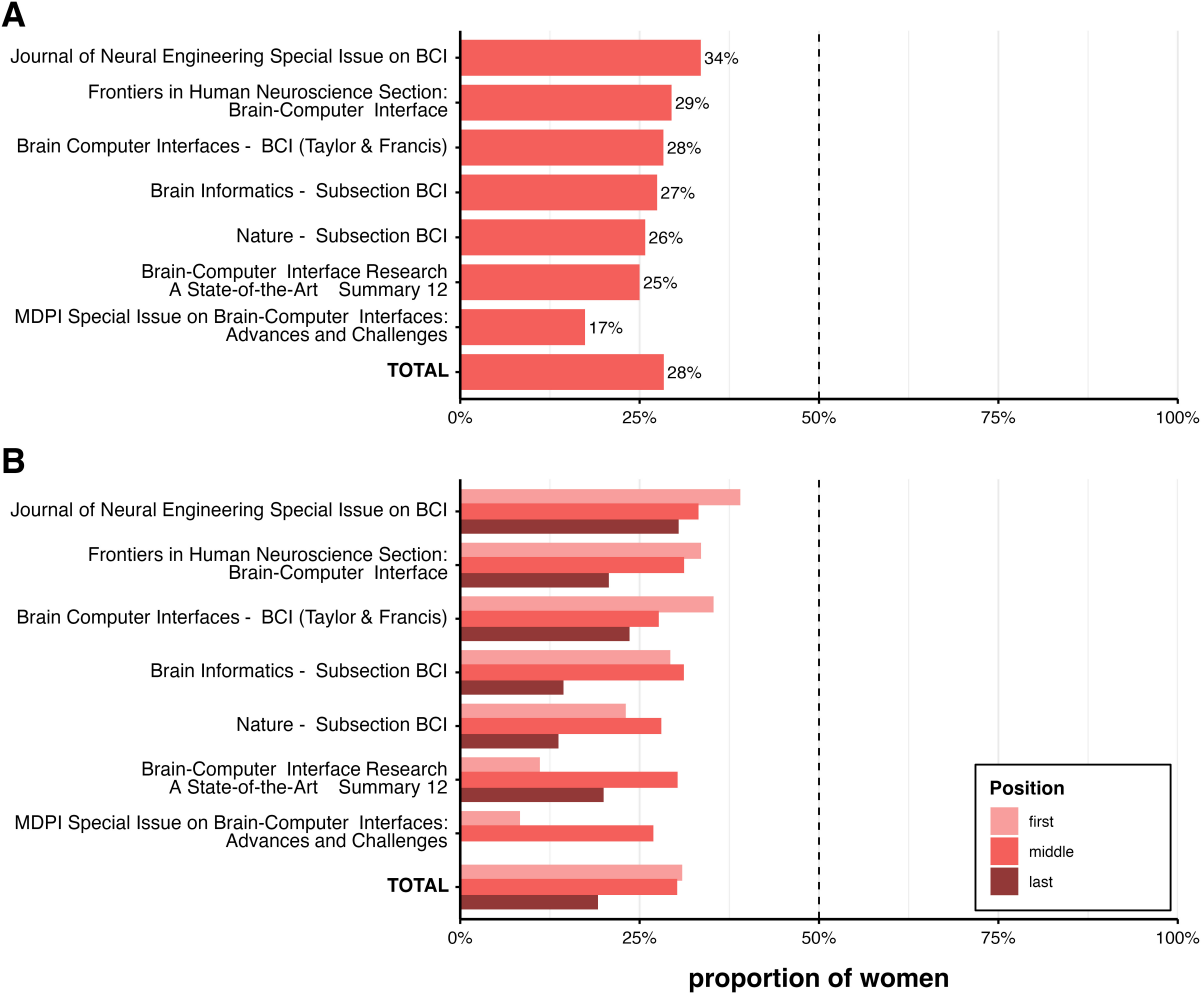
Illustration of the gender distribution among authors publishing in BCI journals, special issues, and book series (upper column **A**) or as first authors, coauthors, or last authors in the BCI journals, special issues, or book series (column **B**), for details, please see the text.

**FIGURE 6:**
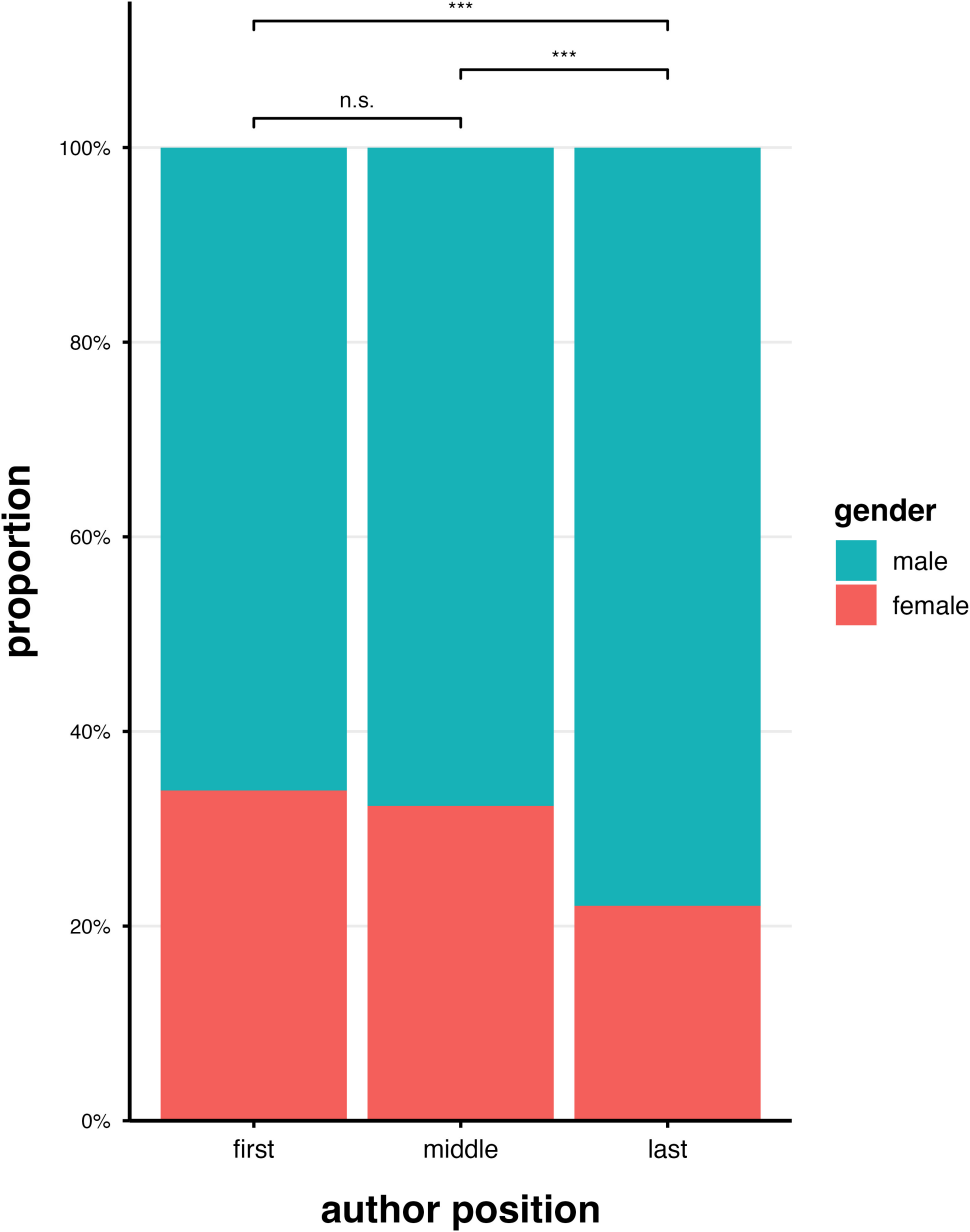
Graphical overview of the statistical results comparing gender distributions of articles published in the BCI journals by first author, coauthor (middle), and last author. *** indicates highly significant.

Overall, we extracted *N* = 827 publications published from 2019 to 2025 (as of July 2025) from all seven BCI publication sources. These publications were authored by a total of *N* = 4.678 authors, of whom 3.693 full names were available after deletion of duplicates (see Figure 4 for an overview). After gender-related preprocessing, 4.312 names (>60% accuracy) or 3.754 names (>80% accuracy) could be identified as belonging to either a woman or a man (Figures 2, 4). As shown in Table 3, the number of women authors was consistently lower than the number of men as authors, regardless of the accuracy threshold of the name identifier (80% or 60%, respectively). The total number of women publishing BCI articles differed significantly from an equal gender distribution of 50% (binomial test, all *p* < 0.001; for an overview, see Table 4a). This also applied to the analyses of authorship positions–whether first author, coauthor, or last author–when examined overall (across all BCI journals) and for each BCI journal individually, as shown in Tables 4b–d. For a visual summary, see Figure 5. Comparisons (chi-square tests of independence) revealed no significant difference in the proportions (women vs. men) across BCI journals, and effects did not differ for first, co-, or last authorships (see Table 5). Among all publications in the BCI journals (including the book series), 30.9% of first authors were women; 30.2% of coauthors were women; and 19.2% of last authors were women. The gender distribution (women versus men) was statistically significant for the roles of the first author, coauthors, and last author, indicating that women are generally underrepresented as authors of BCI publications compared to men, regardless of authorship position. Additionally, the analyses revealed a significant difference in the distribution of women across authorship positions, χ^2^(2) = 34.27, *p* < 0.001: women were more likely to be first authors or coauthors than last authors (see Figure 6 and Tables 6a,b).

**TABLE 3:** Number and percentage of women and men who are authors of publications published in the BCI journals (including the book series).

			>80 threshold			>60 threshold			
	*n* articles	*n* authors	*n* included authors	*n* women	*n* men	*n* included authors	*n* women	*n* men	*n* unknown
Brain Computer Interfaces - BCI (Taylor & Francis)	76	382	346	98	248	360	101	259	15
Frontiers in Human Neuroscience Section: Brain-Computer Interface (Frontiers Media SA)	388	1.967	1.517	447	1.070	1.803	539	1.264	65
Brain Informatics – Subsection BCI (Springer Open)	168	838	656	180	476	770	217	553	28
Nature – Subsection BCI (Springer Nature)	114	961	803	207	596	896	242	654	38
Journal of Neural Engineering Special Issue on BCI (IOP Science)	57	408	334	112	222	370	122	248	25
Special Issue on Brain–Computer Interfaces Advances and Challenges (MDPI, Multidisciplinary Digital Publishing Institute)	12	58	46	8	38	55	10	45	1
Brain-Computer Interface Research - A State-of-the-Art Summary 12 - 2023 (Springer Briefs in Electrical and Computer Engineering)	12	64	52	13	39	58	14	44	4
Total	827	4,678	3,754	1,065	2,689	4,312	1,245	3,067	176

Results are listed according to the accuracy thresholds of the name identifier’s accuracy thresholds (80% in light gray; 60% in dark gray, respectively, see also Figure 2 for an overview).

**TABLE 4a:** Number and percentage of women and men publishing in the BCI journals, including statistical comparisons (binomial tests).

	*N*	*n* women	*n* men	Women [%]	Men [%]	*P*-value	*P*-value after corr.
Brain Computer Interfaces - BCI (Taylor & Francis)	346	98	248	28.32	71.68	*p* < 0.001	*p* < 0.001
Frontiers in Human Neuroscience Section: Brain-Computer Interface (Frontiers Media SA)	1517	447	1070	29.47	70.53	*p* < 0.001	*p* < 0.001
Brain Informatics – Subsection BCI (Springer Open)	656	180	476	27.44	72.56	*p* < 0.001	*p* < 0.001
Nature – Subsection BCI (Springer Nature)	803	207	596	25.78	74.22	*p* < 0.001	*p* < 0.001
Journal of Neural Engineering, Special Issue on BCI (IOP Science)	334	112	222	33.53	66.47	*p* < 0.001	*p* < 0.001
Brain–Computer Interfaces: Advances and Challenges, Special Issue (MDPI, Multidisciplinary Digital Publishing Institute)	46	8	38	17.39	82.61	*p* < 0.001	*p* < 0.001
Brain-Computer Interface Research - A State-of-the-Art Summary 12 – 2023, book series (Springer Briefs in Electrical and Computer Engineering)	52	13	39	25.00	75.00	*p* < 0.001	*p* < 0.001
Total	3,754	1,065	2,689	28.37	71.63	*p* < 0.001	*p* < 0.001

**TABLE 4b:** Number and percentage of women and men publishing as first authors of BCI articles in BCI journals, including statistical comparisons (binomial tests).

	*N*	*n* women	*n* men	Women [%]	Men [%]	*P*-value	*P*-value after corr.
Brain Computer Interfaces - BCI (Taylor & Francis)	68	24	44	35.29	64.71	*p* = 0.021	*p* = 0.027
Frontiers in Human Neuroscience Section: Brain-Computer Interface (Frontiers Media SA)	283	95	188	33.57	66.43	*p* < 0.001	*p* < 0.001
Brain Informatics – Subsection BCI (Springer Open)	123	36	87	29.27	70.73	*p* < 0.001	*p* < 0.001
Nature – Subsection BCI (Springer Nature)	91	21	70	23.08	76.92	*p* < 0.001	*p* < 0.001
Journal of Neural Engineering, Special Issue on BCI (IOP Science)	41	16	25	39.02	60.98	*p* = 0.211	*p* = 0.211
Brain–Computer Interfaces: Advances and Challenges, Special Issue (MDPI, Multidisciplinary Digital Publishing Institute)	12	1	11	8.33	91.67	*p* = 0.006	*p* = 0.0101
Brain-Computer Interface Research - A State-of-the-Art Summary 12 - 2023 (Springer Briefs in Electrical and Computer Engineering)	9	1	8	11.11	88.89	*p* = 0.039	*p* = 0.045
Total	627	194	433	30.94	69.06	*p* < 0.001	*p* < 0.001

**TABLE 4c:** Number and percentage of women and men who are coauthors of BCI articles in BCI journals, including statistical comparisons (binomial tests).

	*N*	*n* women	*n* men	Women [%]	Men [%]	*P*-value	*P*-value after corr.
Brain Computer Interfaces - BCI (Taylor & Francis)	206	57	149	27.67	72.33	*p* < 0.001	*p* < 0.001
Frontiers in Human Neuroscience Section: Brain-Computer Interface (Frontiers Media SA)	920	287	633	31.20	68.80	*p* < 0.001	*p* < 0.001
Brain Informatics – Subsection BCI (Springer Open)	401	125	276	31.17	68.83	*p* < 0.001	*p* < 0.001
Nature – Subsection BCI (Springer Nature)	617	173	444	28.04	71.96	*p* < 0.001	*p* < 0.001
Journal of Neural Engineering, Special Issue on BCI (IOP Science)	247	82	165	33.20	66.80	*p* < 0.001	*p* < 0.001
Special Issue on Brain–Computer Interfaces: Advances and Challenges (MDPI, Multidisciplinary Digital Publishing Institute)	26	7	19	26.92	73.08	*p* = 0.0290	*p* = 0.0331
Brain-Computer Interface Research - A State-of-the-Art Summary 12 - 2023 (Springer Briefs in Electrical and Computer Engineering)	33	10	23	30.30	69.70	*p* = 0.0351	*p* = 0.0351
Total	2,450	741	1,709	30.24	69.76	*p* < 0.001	*p* < 0.001

**TABLE 4d:** Number and percentage of women and men publishing as last authors of BCI articles in BCI journals, including statistical comparisons (binomial tests).

	*N*	*n* women	*n* men	Women [%]	Men [%]	*P*-value	*P*-value after corr.
Brain Computer Interfaces - BCI (Taylor & Francis)	72	17	55	23.61	76.39	*p* < 0.001	*p* < 0.001
Frontiers in Human Neuroscience Section: Brain-Computer Interface (Frontiers Media SA)	314	65	249	20.70	79.30	*p* < 0.001	*p* < 0.001
Brain Informatics – Subsection BCI (Springer Open)	132	19	113	14.39	85.61	*p* < 0.001	*p* < 0.001
Nature – Subsection BCI (Springer Nature)	95	13	82	13.68	86.32	*p* < 0.001	*p* < 0.001
Journal of Neural Engineering, Special Issue on BCI (IOP Science)	46	14	32	30.43	69.57	*p* = 0.011	*p* = 0.013
Special Issue on Brain–Computer Interfaces: Advances and Challenges (MDPI, Multidisciplinary Digital Publishing Institute)	8	0	8	0.00	100.00	*p* = 0.008	*p* = 0.010
Brain-Computer Interface Research - A State-of-the-Art Summary 12 - 2023 (Springer Briefs in Electrical and Computer Engineering)	10	2	8	20.00	80.00	*p* = 0.109	*p* = 0.109
Total	677	130	547	19.20	80.80	*p* < 0.001	*p* < 0.001

**TABLE 5:** Summary of the statistical results comparing gender distributions of articles across BCI journals and for first author, coauthor, and last authorship.

	*P*-value	*P*-value corrected
Chi-Squared-Test	*p* = 0.0815	*p* = 0.146
Fisher’s Exact Test		
First authors	*p* = 0.160	*p* = 0.160
Coauthors	*p* = 0.709	*p* = 0.709
Last authors	*p* = 0.097	*p* = 0.146

**TABLE 6a,b:** Summary of the statistical results (bottom) comparing gender distributions of articles published in the BCI journals (2019–2025) for first author, coauthor, and last authorship (upper column).

Author position	Female	Male	
First	194	433	627
Coauthor	741	1709	2,450
Last	130	547	677
	1065	2689	3754

	***P*-value**	***P*-value corrected**	
Chi-Squared-Test	*p* < 0.001	*p* < 0.001	
*Post hoc* two-proportion *z*-tests			
First vs. co-author	*p* = 0.772	*p* = 0.772	
First vs. last	*p* < 0.001	*p* < 0.001	
Coauthor vs. last	*p* < 0.001	*p* < 0.001	

#### 3.2.1 Potential factors influencing gender and authorship positions in BCI journals

**FIGURE 7:**
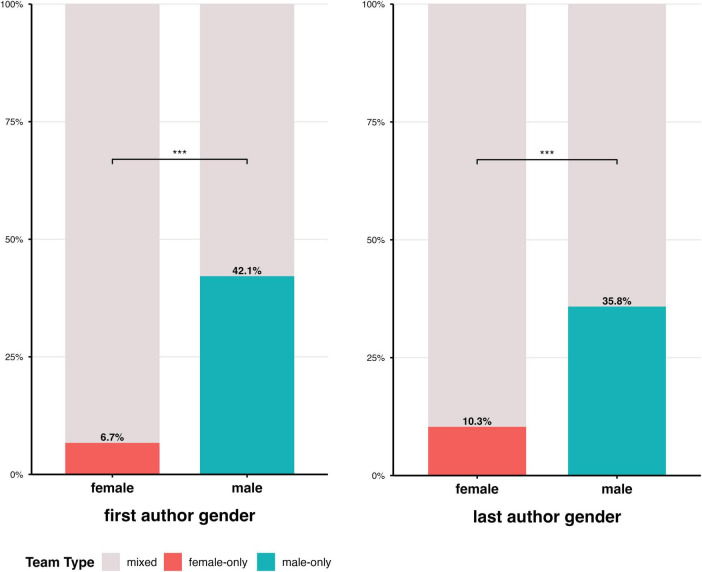
Illustration of gender-based teams of first- and last authors publishing articles in BCI journals in first and last authorship roles.

The analysis of whether women or men published with the same or a different gendered author team revealed significant results. Although this analysis included only a subset of the publications (*N* = 395), significant differences were observed. Of the *N* = 134 articles with a woman as first author, nine (6.7%) were same-gender teams; of the *N* = 261 articles with a man as first author, 110 (42.1%) were same-gender teams, and this difference was statistically significant [two-proportion *z*-test, χ^2^(1) = 51.12, *p* < 0.001]. Among the *N* = 87 articles with a women as last author, 9 (10.3%) were same-gender teams; of the *N* = 307 articles with a male senior author, 110 (35.9%) were same-gender teams, which was also statistically significant (two-proportion *z*-test, χ^2^(1) = 19.70, *p* < 0.001), see Figure 7 and Tables 7a-c for an illustration.

**TABLE 7a–c:** Summary of statistical results comparing gendered teams of authors of articles published in BCI journals for first, co-author, and last authorship.

First author	Same-gender	Mixed	
Woman	9	125	134
Man	110	151	261
	119	276	395

**Last author**	**Same-gender**	**Mixed**	
Woman	9	78	87
Man	110	198	307
	119	276	395

	***P*-value**	***P*-value corrected**	
Two-proportion *z*-tests			
First authors	*p* < 0.001	*p* < 0.001	
Last authors	*p* < 0.001	*p* < 0.001	

**FIGURE 8:**
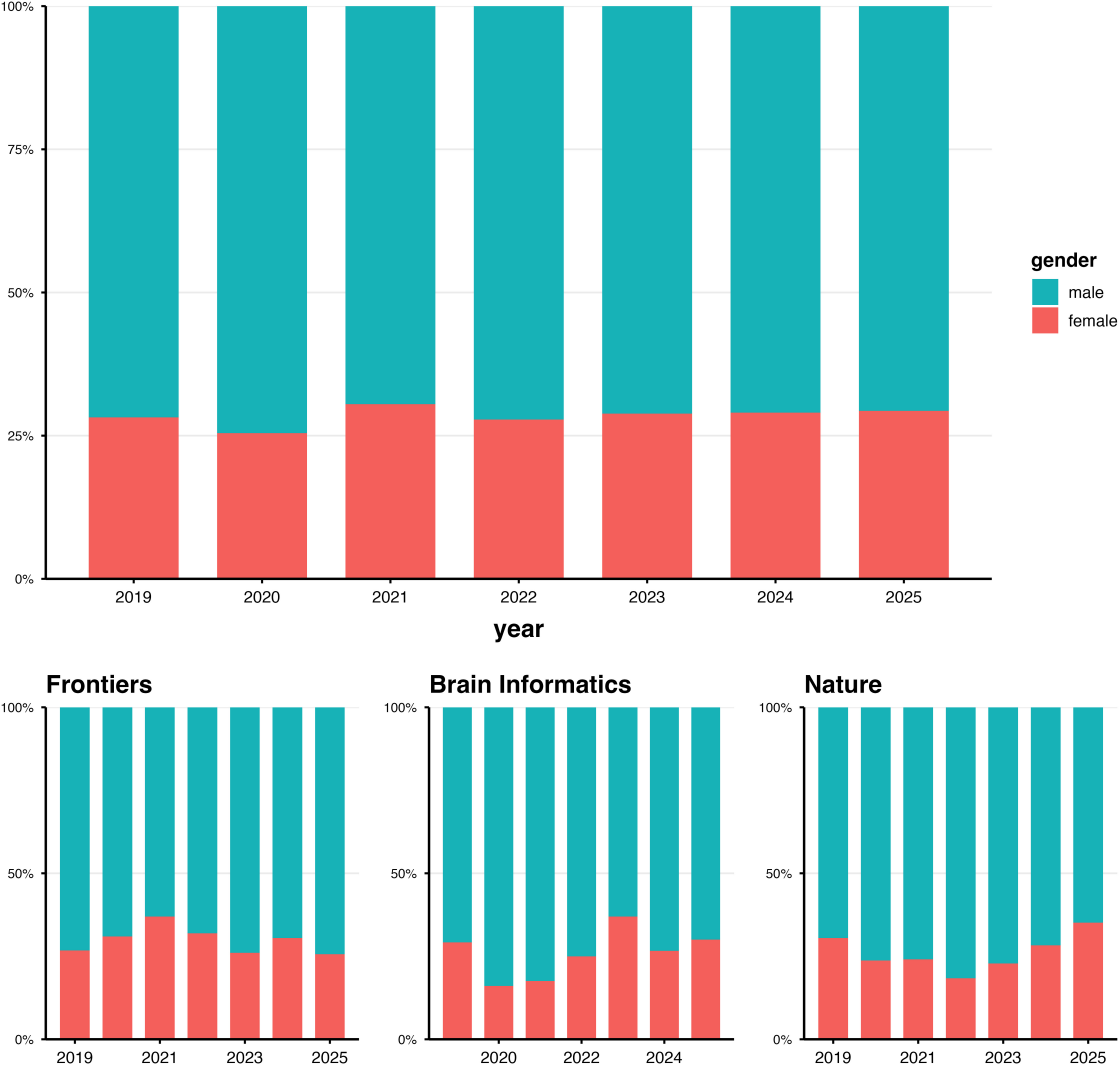
Summary of the statistical results comparing gender distributions of articles published in the three BCI journals with a regular submission rate (2019–2025) over the last 5 years.

The results of the logistic regressions that examined potential factors influencing gender proportions in BCI authorships showed no significant findings, although they revealed several trends. In the regression model that included all authors positions, no significant effects were observed; however, marginal trends indicated lower odds of female authorship in two of the leading BCI journals (Brain Informatics, special section BCI, *p* = 0.0582; Nature, special section BCI, *p* = 0.0623). Additionally, there was a marginally significant interaction between journal and year of publication (*p* = 0.0583 for Brain Informatics × year; *p* = 0.0625 for Nature × year), as shown in Figure 8. When considering only the first author, female authorship was significantly less likely in the special section of one of the leading BCI journals (Nature, special section BCI: β = −892.19, *p* = 0.0381). A significant positive interaction with the year of publication was observed (β = 0.44105, *p* = 0.0381), indicating an increase in female first authorship in the special section of this journal (Nature) over time. Conversely, when analyzing only last authorship, the regression revealed no significant predictors.

### 3.3 Gender biases in BCI publications on psychological human factors and their BCI users

#### 3.3.1 Authors

**FIGURE 9:**
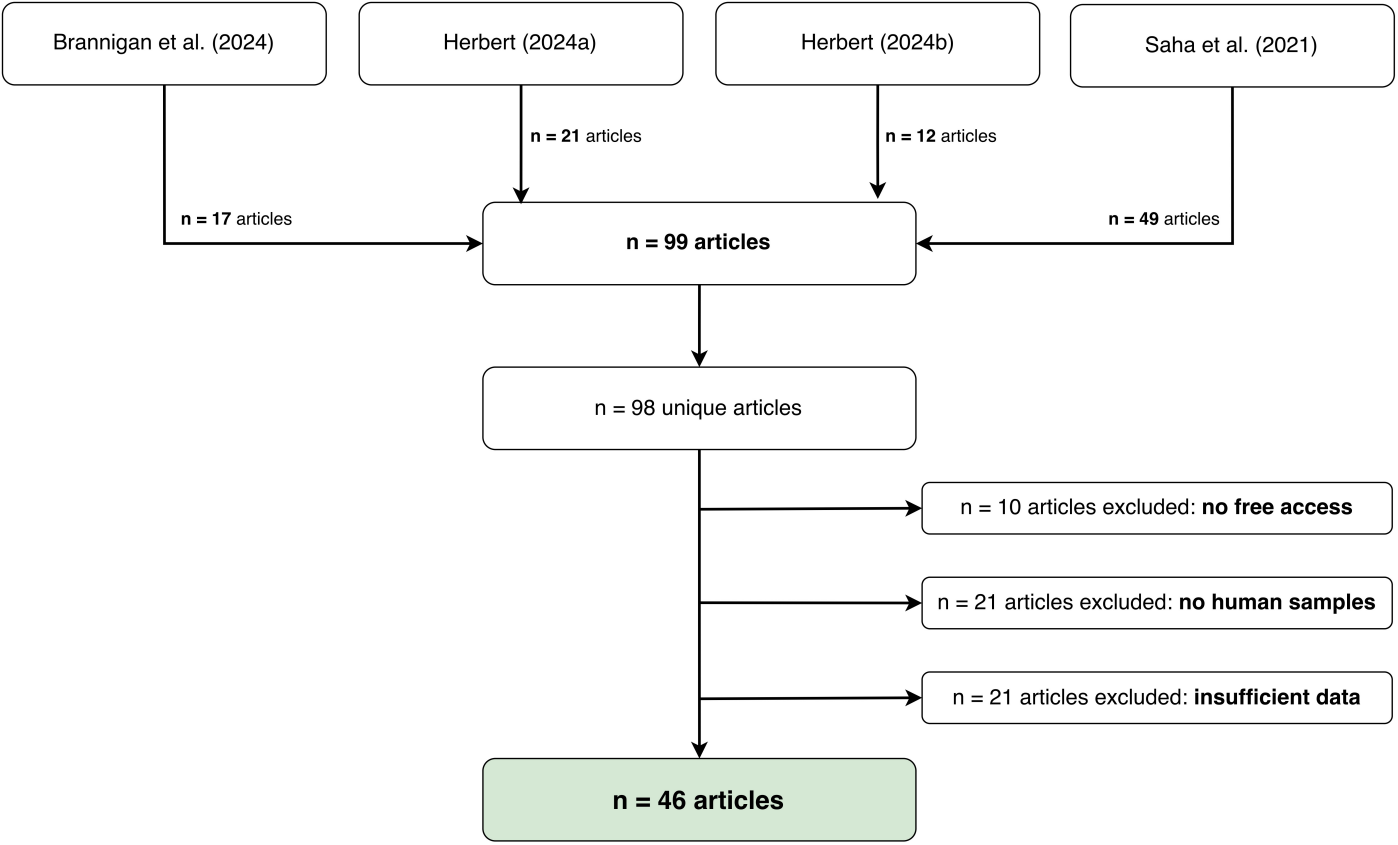
Selection process of the publications taken from the four primary sources Herbert (2024a,b), Saha et al. (2021), and Brannigan et al. (2024).

Analysis of the gender distribution (women versus men) based on the preselected primary publication sources (Brannigan et al., 2024; Herbert, 2024a,b; Saha et al., 2021) showed that 46.48% of first authors, 19.91% of co-authors, and 22.39% of last authors were women. These distributions are significantly different from an equal distribution (binomial tests, all *p*-values < 0.001). As shown in Figure 9 and Tables 8a,b, of the *N* = 98 studies, only *N* = 65 could be included in the analysis due to missing information in *N* = 33 studies that were part of our primary sources (Brannigan et al., 2024; Herbert, 2024a,b; Saha et al., 2021). In the final sample of publications from which all information was available and that examined healthy BCI users, vulnerable BCI users, or mixed user groups (including vulnerable groups and healthy control groups), the percentage of women as authors was consistently lower than that of men. This difference was statistically significant in studies focusing on healthy users (see tables below). To prevent publication biases in the four primary sources (Brannigan et al., 2024; Herbert, 2024a,b; Saha et al., 2021), Fisher’s exact test was conducted, which showed no significant difference between the three types of studies [healthy participants, vulnerable groups, or healthy and vulnerable participant groups (*p* = 0.200)].

**TABLE 8a,b:** Summary of the statistical results comparing the gender distributions of authors publishing studies on the role of psychological human factors on BCI performance.

		*n* women	*n* men	Women proportion [%]	Men proportion [%]	*P*-value	Adjusted *P*-value
First	33	38	46.48	53.52	*p* < 0.001	*p* < 0.001	
Coauthor	67	282	19.20	80.80	*p* < 0.001	*p* < 0.001	
Last	15	52	22.39	77.61	*p* < 0.001	*p* < 0.001	

**Type of study**	***n* articles**	***n* women**	***n* men**	**Women proportion [%]**	**Men proportion [%]**	***P*-value**	**Adjusted *P*-value**
Healthy	40	30	101	22.09	77.01	*p* < 0.001	*p* < 0.001
Mixed	3	6	10	37.05	62.05	*p* = 0.754	*p* = 0.754
Non- healthy	22	44	98	30.99	69.01	*p* = 0.363	*p* = 0.545

#### 3.3.2 Gender biases and BCI users

**FIGURE 10:**
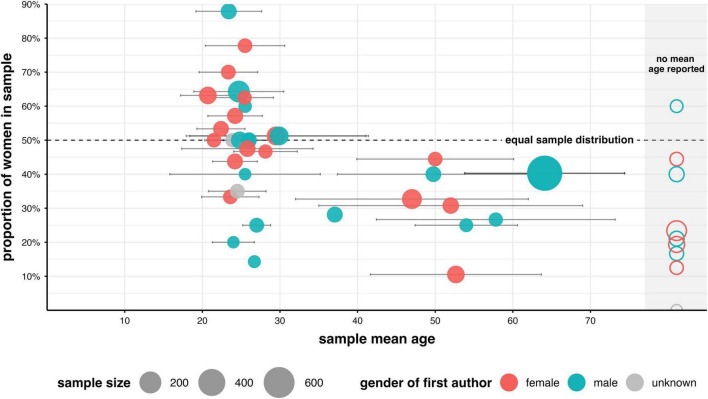
Distribution of studies by sample size, gender, and – where available – age distribution.

Analysis of the participant samples in the selected publications showed that 61.54% of the publications examined only healthy BCI users, 33.85% investigated only patient samples, and 4.62% reported investigating both patient samples and healthy samples. The studies showed a wide range of sample sizes (see Table 9). Some studies had only very few participants (*n* = 2). Seventy-three studies reported their users’ ages. Across studies, the mean age of the BCI users varied significantly (Kruskal-Wallis test, χ^2^(2) = 16.75, *p* < 0.001). For the studies involving healthy participants, the mean age was 26.88 years (SD = 6.12). In the patient studies, the mean age was 51.20 years (SD = 11.94). *Post-hoc* tests (Dunn tests for pairwise comparisons) revealed a significant difference in age between studies that included healthy participants and those that included patient samples. However, this might be due to the relatively small number of publications that used mixed samples (see Tables 9a,b). Forty-six studies reported the sample size and the gender distribution of their participants in more detail (see Figure 9). Among these studies, five had equal gender distribution, twelve had more female participants, and twenty-nine had more male participants. In eight of these studies (17.4%), the gender distribution significantly differed from equal in binomial tests – three with more women and five with more men. Overall, the *N* = 46 studies included 1,213 women and 1,673 men, indicating a significant imbalance in the number of women and men as volunteers (binomial test, *p* < 0.001). As shown in Figure 10, descriptively, some studies examining samples from older age groups favored men as participants and had male first authors. Studies examining samples with younger participants included a relatively balanced gender distribution. However, across all samples, the studies included a higher proportion of men. This could be due to bias in sampling from primary sources (Chamberlain et al., 2022; Glim et al., 2023; Jausovec and Jausovec, 2010; Wickham, 2024), in study designs, or in the types of disorders among participants in studies examining elderly BCI users.

**TABLE 9a,b:** Summary of the statistical results comparing the age distributions of the participants volunteering for the BCI studies. For studies with mixed samples, we calculated weighted mean age scores.

Type of participants	*N*	*N* with age reported	Mean age (years)	SD (years)
Healthy	39	27	26.88	6.12
Mixed	3	2	43.55	20.16
Non-healthy	22	8	51.20	11.94

		***P*-value**	***P*-value corrected**	
Kruskal-Wallis-Test		*p* < 0.001	*p* < 0.001	
*Post hoc* Dunn-Tests				
Healthy vs. mixed		*p* = 0.060	*p* = 0.18	
Healthy vs. non-healthy		*p* < 0.001	*p* < 0.001	
Mixed vs. non-healthy		*p* = 0.832	*p* = 0.832	

#### 3.3.3 Relationship between the gender of the author and the BCI users

We also examined the relationship between participants’ gender and the authors’ gender who published the study. Of the forty-six included studies, only twenty had a woman as the first author. Six of these studies demonstrated a significant gender bias in their participant samples. Among the sixteen studies with male first authors, six also showed a significant gender bias. For three studies, the gender of the first author was unknown, and one of these showed a considerable bias. This information is detailed in Table 10. However, according to statistical testing (Fisher’s exact test), no significant difference was found among the distributions, suggesting that the gender of first authors was not associated with substantial gender biases in the participant samples (*p* = 0.6213). A detailed overview of all studies extracted from the primary sources (Brannigan et al., 2024; Herbert, 2024a,b; Saha et al., 2021) is provided in Table 2.

**TABLE 10:** Relationship between the gender of the author and the gender of the BCI user.

First author	Significant difference (*p* < 0.05)	n.s. difference (*p* > 0.05)
Woman	16	4
Man	19	3
Unknown	3	1

### 3.4 Gender- and sex differences in neural processing relevant for BCI

Table 11 and the Supplementary Table 1 summarizes all hits from both broad and narrow key term searches, after manual approval of the inclusion and exclusion criteria (see Section “2.1.3 Gender distribution in BCI publications on psychological human factors and their users”). After careful evaluation, fifty-four studies (*N* = 54) met the inclusion criteria (see Table 11 and the Supplementary Table 1). These publications qualified as studies reporting gender- or sex differences in neural processing recorded with non-invasive EEG methods. Hits from the broad search included reviews (R), research articles (A), and three conference papers (CP). In contrast, only one study was identified with the narrow search that combined analysis across BCI data sets (D). Two additional BCI studies that did not fully meet the search criteria, as indicated by the title, but did analyze gender effects on SSVEP-BCI performance, are also listed in Table 11; see references 2 and 3 in Table 11. Of these, only one study, which reported preliminary results from a conference publication, found better BCI performance parameters among women. In contrast, the second study could not replicate this gender effect using the same SSVEP-BCI (see [2-3] in Table 11).

**TABLE 11:** Comprehensive summary of all eligible studies from the literature search in the publication period from 1980 to 2025.

Ref	Type	Year	Title	Participants (*N*)	Number women (*N*)	Number men (*N*)	Healthy participants vs. patient groups	Age of participants	Method	Abstract summary	Gender- or sex-differences	Gender- or sex-differences observed	Results
Specific Search: EEG & BCI & gender/sex differences (for details see Figure 1)													
For the full table, including all hits of the literature review details, see Supplementary Table 1													
Broad Search: EEG & gender/sex differences (for details see Figure 1)													
For the full table, including all hits of the literature review details, see Supplementary Table 1													

Hits include the following article types: reviews (R), research articles (A), and multisite studies combining analyses across datasets (D). For the full table, including all hits of the literature review, please see the Supplementary Table 1.

**FIGURE 11:**
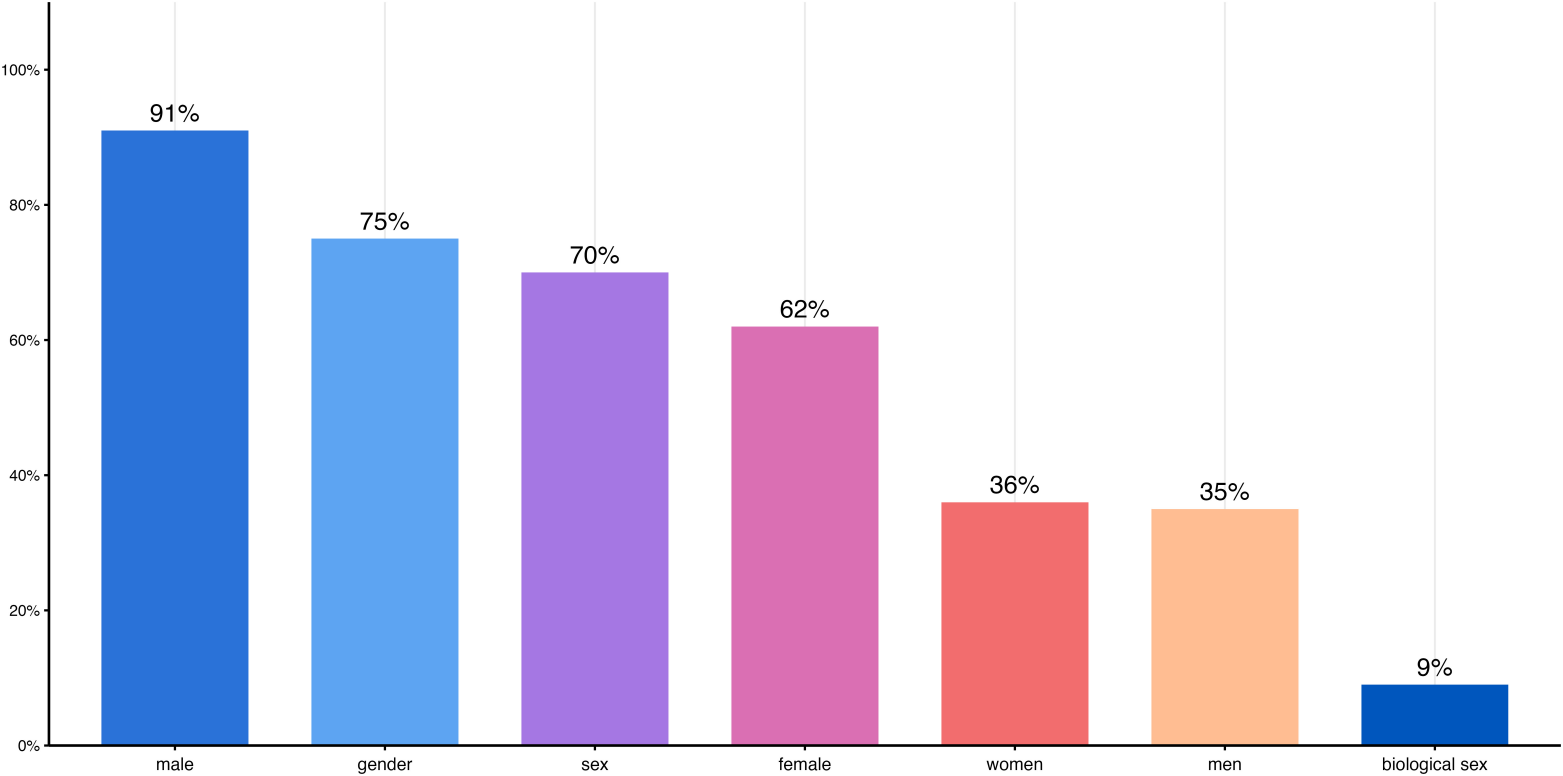
Frequency of the terms “male,” “female,” “men,” “women,” “gender,” and “sex” detected by MAXQDA.

In total, most studies reported the number of women and men included in their study samples in the manuscript. Among the BCI studies, however, only one did so. As shown in Table 11, gender- or sex-related differences were investigated across a wide range of experimental conditions and electrophysiological markers relevant to BCI research. These experimental conditions included EEG resting states, altered states of consciousness such as sleep and pain, the effects of specific drugs or medications, and the effects of neurological or mental disorders on neural processing. The studies also examined gender- or sex-related differences in specific experimental task conditions, including cognitive and affective processing, spatial navigation training, and action observation. Most studies focused on gender- or sex-related differences in EEG frequencies or EEG-ERPs, whereas others examined more specific EEG markers, such as EEG coherence, connectivity, or microstates. Almost all publications listed in Table 11 and Supplementary Table 1 from the broad search reported gender- or sex-related differences in their outcome measures. This contrasts with the findings from the publications identified through the targeted search strategy, which focused explicitly on BCI studies. The detailed in-depth search suggests that most studies reported the gender or sex of their participants in the manuscript (see Table 11 and the Supplementary Table 1). Of *N* = 54 studies, *N* = 37 reported participants’ gender, *N* = 17 reported participants’ sex, and one study did not specify or report either. Among all publications, the terms “gender” and “sex” were used most often in titles or abstracts, whereas the terms “female(s)” and “male(s)” appeared more frequently than “women” and “men,” as shown in Figure 11.

## 4 Discussion

Research on Brain-Computer Interfaces (BCIs) is rapidly growing, with a wide range of multidisciplinary applications. This study examined the science behind BCIs and explored the gender distributions of editorial boards, scientists publishing BCI research, and participants volunteering for BCI studies. Moreover, a review was included that explored potential sex- and gender-related differences relevant for BCI research. Our findings suggest that most scientists on editorial boards and authoring BCI publications in BCI journals are men. The gender ratio in BCI studies, however, can vary widely. Gender- or sex-related differences in EEG brain signals have been reported in the literature. The review indicates that further systematic investigation is still needed regarding effects of gender and sex on BCI performance.

### 4.1 Gender and authorship

Considering all articles published in the past 5 years that were eligible for an analysis after gender preprocessing of author names (*N* = 3754), we found a gender ratio of approximately 28:71 (women to men). This included journals that focused explicitly on BCI research as well as those with BCIs subsections that published special issues dedicated to BCIs. Additionally, a book series representing various roles in academic careers, from early-career scientists to senior researchers, was included. Overall, a 30:70 (women to men) ratio was observed for first authors and coauthors, decreasing to 20:80 (women to men) for the last author position. Taken together, the analysis suggests that women as authors are generally in the minority and less likely than men to publish as last authors. The highest and lowest ratios–39:61 for women to men as first authors and zero women as authors–were observed in the special issues (see Figure 6 and Table 4). For BCI journals with a regular publication rate, the ratio did not differ significantly between journals.

Regular assessments of the number of women and men in science consistently show that still only 30% of researchers worldwide are women; see Introduction. This gap is especially pronounced in scientific fields such as the natural sciences, technology, engineering, and mathematics (STEM). However, the 30% women-to-70% men ratio is also observed in many other disciplines, including medicine, the social sciences, and the arts (STEAM) (Card et al., 2023; Casad et al., 2021; Holman et al., 2018; Larivière et al., 2013; Llorens et al., 2021). According to the literature (Card et al., 2023; Casad et al., 2021; Holman et al., 2018; Larivière et al., 2013; Llorens et al., 2021; Odic and Wojcik, 2020), the 30:70 trend persists even in disciplines where women usually represent a much higher percentage of student populations, such as medicine and psychology, which are also heavily involved in and contribute to Brain-Computer Interface (BCI) research.

Our second analysis, which focused on BCI studies examining psychological human factors and BCI user performance, reflects those specialized areas. However, this analysis revealed trends similar to those observed in the analysis involving authors publishing BCI articles in BCI journals overall. Although the ratio of women to men as first authors was higher in these BCI studies (47% women, 53% men), the overall distribution of women and men as authors remained significantly unequal. Additionally, only 20% of coauthors were women, and about 22% of last authors were women. This reflected the trends observed in the analysis, which included all articles from BCI journals, regardless of their topic (see Tables 4, 8 for comparisons). The analysis of authors in psychological human factor BCI studies included *N* = 98 articles, taken from four primary references that summarized BCI publications examining BCI performance in the most common BCIs [P300 BCI and SMR-BCI (Herbert, 2024a; Saha et al., 2021), or SSVEP-BCI (Saha et al., 2021)], newer BCI developments [affective BCIs and linguistic BCIs (Herbert, 2024b)], and BCI usability (Brannigan et al., 2024) among healthy and patient groups. Of these studies, *N* = 65 studies provided complete data for analysis. To identify and control for potential publication biases, we also examined the impact of the research topic specified by the primary source and the type of study, whether it focused solely on healthy users or clinical patient groups. The results of this analysis suggested that the percentage of women as authors was lower than that of men across all studies, and this disparity was particularly pronounced for the first author role in studies examining healthy users (see Tables 8a,b).

Taken together, the results on authorship in BCI publications confirm an imbalance between women and men in authorship that has been observed worldwide in the scientific community. The results support this trend within the field of BCI, even in studies focusing on psychological human factors, BCI performance, or BCI usability, which represent a BCI domain with a broader multidisciplinary background. The reasons for the underrepresentation of women as authors in BCI studies require a more systematic investigation to confirm the findings before any interpretation beyond speculation can be made.

Additionally, another observation warrants attention and further careful analysis. The exploration of gender relations among authors publishing BCI articles in BCI journals revealed that men are more likely than women to publish with same-gender teams, whether as first or last author. 42.1% of articles with men as first authors and 35.9% with men as last authors involved teams of the same gender (first, co-, or last author sharing the same gender). For women as the last author, only 10.3% of publications involved same-gender teams, and 6.7% when women were the first authors. This difference in publication bias was significant, suggesting that it might occur more often with men than with women as first or last authors. These findings confirm a growing trend in gender-biased publishing preferences, suggesting that men tend to co-publish more frequently with men than with women, and vice versa (Holman and Morandin, 2019; Salerno et al., 2019). Previous studies have indicated that gender-biased publishing can serve as an indicator of collaborative academic interactions between women and men (Holman and Morandin, 2019; Salerno et al., 2019). Gender-biased publication trends might promote gender homophily (Zhou et al., 2024), and widen the gender gap in academia, reduce openness and transparency regarding gender-related aspects in authorship, and hinder gender-diverse teams, which have been shown to produce more novel and innovative research results than same-gender teams (Mattoon et al., 2024; Yang et al., 2022). However, these assumptions and our results should be treated with caution. Similar to previous research, our analyses rely on a collection of statistical names, their gender classifications, and the authorship positions associated with them. We did not make specific adjustments for the observed gender ratios (fewer women than men), nor did we examine the authors’ affiliations, academic roles, or the broader academic network and positions. Therefore, the finding of same-gender effects in publications should not be overemphasized or generalized. However, further exploration of gender-biased publication effects could be valuable in future studies. These studies could categorize publications within larger, multisite, and interdisciplinary projects, where collaborations and authors’ academic roles are clearly documented, publicly accessible, and well-defined.

There is evidence that the COVID-19 pandemic has exacerbated the existing gender gap in academia by affecting the research productivity of both men and women, as well as the number of articles published (Lee et al., 2023; Ribarovska et al., 2021). Some disciplines have been more affected than others. The current analysis of BCI authorship in publications from the past 5 years indicates that the observed gender imbalance was consistent and minimally affected by publication year, despite the COVID-19 pandemic occurring during this period. Logistic regressions, however, revealed specific trends that, in follow-up, showed a significant decrease in the likelihood of being a first author and a woman in the special section of one of the leading high-impact journals with a BCI subsection (see Figure 8). Using the year of publication as a predictor revealed an increase in first authorships for women in this journal over time, whereas last authorship remained stable. It remains unclear whether the lack of gender parity in authorship of BCI publications and its consistency over the past 5 years are due to self-selection, lack of support, incentives, motivation, or gender-related social barriers. This question remains to be explored. Nevertheless, our observations suggest that gender effects in the BCI literature are evident and require further research to understand their causes. This also relates to the studies on psychological human factors and BCI performance and BCI usability discussed next.

### 4.2 Gender and BCI participant samples

BCI studies on psychological human factors and BCI performance, or BCI usability, had a higher proportion of men as participants. Moreover, especially those involving older participants, were authored mainly by men as first authors. This aligns with previous findings that the experimenter’s and participants’ genders can influence each other and may affect BCI performance (Chapman et al., 2018). BCI performance in the SMR-BCI, for example, was previously found to be better in studies in which women served as experimenters (Pillette et al., 2021). As shown in Figure 8 and summarized in Table 10, the potential relationships among participants’ age, status (healthy, patients, or controls), gender, and the first author’s gender warrant further examination. Furthermore, data preprocessing revealed that over half of the selected *N* = 98 studies could not be included in the analysis due to missing information on the participants’ gender, age, or health status. Additionally, most studies with larger sample sizes and more men as participants were survey studies on BCI usability among patients. This is important because differences in BCI usability have been reported in the literature (El-Osta et al., 2025; Pasqualotto et al., 2011). Moreover, previous research has indicated significant variations in attitudes, trust, and motivation to use technology as an aid or device across individual user profiles, including differences between men and women (Cai et al., 2017). Women and men may also have different concerns about privacy and data security, especially in studies involving health technology (Carbone et al., 2024; Wilkowska and Ziefle, 2011). These factors could influence women’s and men’s willingness to participate, their motivation to perform well, and their beliefs about their ability to use the BCI effectively. As pointed out in Brannigan et al. (2024), achieving satisfactory speed and accuracy in BCI performance still requires a high level of training that exceeds the capabilities of most patients. Our review of the studies suggests that research on BCI usability may be biased. Therefore, it is crucial to consistently report participants’ gender in BCI usability studies, as the gender distribution appears uneven across previous usability studies that have investigated patients as users (see Table 2).

It has also been suggested that women and men are represented differently in specific clinical trials and have different motivations for participating in scientific studies (Wilkowska and Ziefle, 2011). As shown in Table 2, only three studies from the primary reference sources (Herbert, 2024a,b; Saha et al., 2021) that investigated EEG-BCI performance reported equal numbers of men and women. Twenty-one publications reported participant samples with a higher proportion of men than women. Twelve publications reported more women than men as participants. However, among studies with healthy, young participants, most had more women than men (see Table 2). Methods used to recruit healthy participants and the disciplines conducting the research can contribute to gender biases. The gender distribution among student populations might not reflect that of the larger community. Although our analyses are limited to studies included in the primary reference sources (Brannigan et al., 2024; Herbert, 2024a,b; Saha et al., 2021) and therefore should not be overgeneralized, they highlight the importance of inclusive and representative research practices in BCI studies. In theory, a complex interaction of cultural, social, and methodological factors could influence the gender distribution in scientific studies and BCI performance [see (Herbert, 2024b) for a discussion].

### 4.3 Gender and editorial boards

Gender effects can also influence editorial boards that determine the quality of studies and their acceptance for publication, as well as the research topics that dominate the field. Compared to the analysis of BCI authorship, the review of editorial boards revealed an even smaller number of women in editorial roles. Overall, only 28% of editors in the BCI journals, including those with regular publications, special issues, or book series, were women. The highest gender ratio achieved was 80:20, favoring women as editors. This ratio was achieved in a special issue, similar to the book series, which published work from the 2023 International BCI Meeting, highlighting research by young investigators and their senior teams. In contrast, only 14.29% of the editors in the book series were women. The lowest gender ratio, with 0% women and 100% men as editors, was found in the special issue on advances and challenges in BCI research (see Table 1b). Typically, guest editors are responsible for handling special issues. Therefore, the findings suggest that the influence of women and men as potential contributors to scientific topics can be ambiguous. Moreover, the variability in gender distributions suggests that having women as editors in BCI special issue research does not necessarily imply more women than men as authors in these special issues. The gender distribution of authors publishing in the special issue, which had a high proportion of women as editors, was 33.53% women and 66.47% men. Although this ratio reflects the typical gender ratio reported in the literature, it was still lower for women in the special issue, which had no women as editors. The BCI journal, which regularly publishes articles and has the highest proportion of women in editorial roles, with a nearly 58:42 women-to-men ratio, also achieved the highest impact score. However, similar to the special issues with a higher proportion of women as editors, the gender ratio of authors publishing in it was 26% women to 74% men, which is slightly lower than the typical 30:70 gender ratio reported in the literature and lower than the ratio in authorship achieved by the other BCI journals. The analysis of editorial boards of leading neuroscientific journals that are open but not exclusive to BCI research revealed a similar pattern: women were significantly underrepresented in editor roles compared to men. However, descriptively, three neuroscientific flagship journals included in the analysis (see Table 1a) displayed a higher proportion of women; two of these journals primarily focus on psychology and related disciplines investigating psychophysiological mind-body relationships. Whether these examples are representative of or best practices for promoting women in influential roles, such as editorial board membership in scientific fields traditionally dominated by men, would be an interesting area for future research. For example, one of the journals that exceeded the 30:70 ratio in editorial boards is supported by initiatives from a diversity and outreach committee. The committee’s goal is to promote diversity, inclusion, and gender equity by reducing gender disparities and supporting women and gender minorities in psychophysiology.^7^ Notably, the BCI journal with the highest impact factor also established similar boards^8^.

### 4.4 Gender- and sex differences in EEG brain signals

Finally, the exploratory literature review suggests that considering participants’ gender and sex may be an important factor in BCI studies. As shown in Table 11 and the Supplementary Table 1, future research would benefit from more organized study designs and methods for gender- and sex-related differences in electrophysiological signal recording and analysis. The studies selected from the literature search indicated that several factors may explain why EEG indicators relevant to BCI research differ between women and men. These include differences in various tasks, as well as mental, affective, or cognitive processing, and processing strategies. Additionally, there may be hemispheric variations or different responses during states of resting brain activity, sleep, altered consciousness, medication, or mental and neurological disorders. Moreover, electrophysiological indicators of neural processing could be influenced by social and cultural norms associated with gender biases. This also applies to specific EEG signals, such as the P300 and mu-rhythm variations, which are often used as BCI classifiers in previous research, including the studies summarized in Table 11 and Supplementary Table 1. The studies have found that women exhibit more pronounced mu suppression when observing hand actions than men do, and some EEG studies report gender- and sex-related differences in mu-rhythm activity linked to motor imagery. This suggests possible differences between women and men in how the brain processes actions relevant to BCI applications.

Therefore, gender- or sex differences, if unrecognized, could implicitly influence BCI performance, BCI accuracy, and BCI classification. According to previous research, approximately 15%–30% of BCI users fail to achieve the accuracy necessary for BCI classifiers to predict the user’s behavior correctly (Allison and Neuper, 2010; Blankertz et al., 2009). BCI illiteracy can manifest as an inability to use a BCI for its intended purpose accurately. However, as revealed by our narrower search, recent publications found no indication that the phenomenon of BCI illiteracy or BCI usability is related to gender or sex. However, as shown in the Supplementary Table 1, only one study was found that focused on gender effects for brain signals associated specifically with BCI performance (P300 and mu-rhythm) (see the Supplementary Table 1, first line, reference 1). The study evaluated different data sets taken from SMR-BCI studies and gender differences in my-rhythm activity. To clarify the influence of gender and sex on BCI performance, the authors combined four SMR-BCI datasets based on motor imagery tasks into a single, large dataset comprising *N* = 248 participants and a balanced gender distribution. The results showed no significant difference in the mu suppression index between women and men as BCI users (see the Supplementary Table 1). However, two recent conference publications that did not qualify for hits with the narrow search terms (see Figures 1a,b) suggest opposite findings. The results indicate that EEG signals of women elicited during motor imagery could be identified with higher accuracy by traditional machine learning algorithms than those of men. Moreover, women showed better control of motor imagery BCI than men (Alimardani and Gherman, 2022; Wang et al., 2023). Two further publications that focused on gender as a critical demographic variable in BCIs reported similarly controversial findings. While gender effects were observed in an earlier study of SSVEP BCIs, a more detailed analysis of the data failed to reproduce this finding (see Supplementary Table 1, references 2-3).

Taken together, this suggests that gender- and sex-related differences in neural processing should not be ignored in BCI studies. Further research is needed to develop a more systematic BCI approach. The exploratory review of the EEG literature on gender and sex differences provides a first step in this direction. It shows that previous research on electrophysiological brain signals during rest, altered states of consciousness, medication, mental disorders, or psychological tasks is quite inconsistent and lacks a rigorous research approach, standard methodology, normative data, and uniform study designs. Examining gender- and sex differences in EEG studies and BCI research could benefit from more detailed investigations, such as reviews and meta-analyses, that validate how women and men as BCI users perceive, process, and interpret specific stimuli and perform particular tasks. This would help clarify the variability both within and between individuals in EEG brain signals used for BCI research and training.

## 5 Strengths, limitations, and outlook

This study included a systematic analysis of gender distributions among academic roles and participant status, supported by a exploratory review of the existing literature on gender- and sex-related differences in EEG signals relevant to BCI studies. This comprehensive, multilevel analysis is a key strength of the present study, advancing previous research and shedding further light on the role of gender in academic research.

Methodologically, the study used validated software tools to automatically analyze the gender of first names for editorial board members and authors, thereby enhancing transparency and reliability in data preprocessing. However, such tools have certain limitations. In this study, we used the well-established Gender-Api, which is considered a validated option with a low error rate (Santamaría and Mihaljević, 2018; Sebo, 2021; VanHelene et al., 2024). A disadvantage of the tool is its accuracy when assessing names from Western versus Eastern cultures. Names that are common in Western regions, such as Europe and America, are recognized with high accuracy. Names from East Asian countries tend to have significantly lower accuracy rates (Sebo, 2021; VanHelene et al., 2024). BCI is a global research area with scientists worldwide. Research shows (VanHelene et al., 2024) that the accuracy of gender tools, such as the one used here, is only 82% or lower for names typical of authors from Asian countries. Gender identification tools may introduce cultural biases, especially for less familiar names. Reports, such as the UNESCO study from 2021, indicate that gender biases vary widely across countries and are most pronounced in G20 nations, while they are less severe in Central Asian countries, which have some of the highest proportions of women researchers^9^.

Additionally, some names may be classified as unisex or non-binary, while others may have different gender associations across countries. This could lead to misclassification if gender is not verified in person. In the current study, we tested the reliability of the gender tool using conservative (80%) and lenient (60%) accuracy thresholds (see Table 1d). The results were consistent, suggesting that the datasets used in this study were less diverse. In addition, the analysis of authorship status needs attention. We examined the frequency with which women and men are listed as first, co-, and last authors of BCI publications. Being listed as the last author doesn’t necessarily imply holding a senior position or being a senior scientist, as discussed in detail in the section “2 Materials and methods.” Future research could expand the analyses by verifying authorship roles using additional data, such as affiliations, correspondence, or authorship contributions reported in some but not all papers, depending on the journal’s guidelines. Moreover, future research should extend the analysis to culture as an essential additional factor affecting both women’s and men’s roles in the scientific process.

Last but not least, the analysis of the participants’ gender and the literature review of gender- and sex-related differences in neural processing revealed the need for greater transparency. Among the selected articles focusing on BCI performance, nearly half did not report participants’ gender. This highlights the need for guidelines on how gender or sex should be reported in BCI studies. Descriptively, gender was unevenly distributed across participant samples: BCI studies that included patient samples and older age groups tended to include more men than women. Moreover, we explored whether subtle associations with the authors’ gender could be observed. Our analysis could not determine the factors that influenced the ratio of women to men volunteering as BCI users, such as whether gender bias in the disorders of the patients, different self-selection by women and men to participate in BCI studies, or experimenter effects observed in previous clinical and experimental research, including BCI studies, played a role. However, since gender- and sex differences in neural correlates have been reported in many studies in our exploratory review, and because experimenter and self-selection effects in scientific studies are gender-sensitive, future BCI research should pay closer attention to these factors. For example, conducting regular meta-analyses or review studies could help identify gaps in experimental research and establish standards for gender-sensitive BCI studies that also consider cultural factors.

In the present study, gender was estimated from the authors’ and editors’ first names and thus reflects a binary classification (man/woman). This classification excludes non-binary and other gender identities. Future research could explore beyond the binary gender. Gender and sex encompass more than just binary categories. In the literature review, studies on sex and gender differences were included. The analysis showed that gender and sex were mentioned most often (see Figure 11). The term “biological sex” was used rarely, and studies preferred using the terms’ male(s)’ and “female(s)” instead of “women” and “men.” Additionally, some studies lacked age and some gender/sex information in the abstracts. Abstracts serve as concise, standalone summaries of research papers, enabling readers to quickly understand the study’s purpose, methodology, key findings, and conclusions. We recommend that future studies on gender- and sex-related differences, in general and in BCI research in particular, include as much information as possible in the abstract and title. This will promote comparability of research findings.

## 6 Conclusion

Despite its progress, BCI research can be influenced by gender biases. These occur among editorial members, researchers publishing studies, and even participants involved in the research. Further research should aim to assess potential causes of these biases and evaluate interventions to address them. Brain signals obtained from non-invasive EEG methods can vary between men and women due to factors such as age, mental processing, medication, and specific disorders. Therefore, future BCI studies should include information on the participants’ age, gender, and the methods used to collect this information. This information is important for any BCI modalities, training methods, usability, and performance outcomes to meet the specific needs of the users. Further systematic research is necessary to ethically investigate these sensitive and individualized factors. Understanding gender, sex, and user-related characteristics in BCI studies will deepen our understanding of BCI users and their unique preferences and needs. In line with current endeavors emphasizing the importance of addressing culture, language, and demographic variables representatively in BCI research (Herbert, 2024b), recommendations should be provided for effectively integrating gender-related topics and sensitive information, such as participant sex, into BCI research.

## Data Availability

The original contributions presented in this study are included in this article/Supplementary material, further inquiries can be directed to the corresponding author.

## Footnotes

1
https://www.bci-award.com/Books

2 MDPI Special Issue Brain–Computer Interfaces (BCI) and Applications in Healthy and Daily Life Activities was not included in the analysis for the reasons provided in the text.

3
https://www.crossref.org/documentation/retrieve-metadata/rest-api/

4
https://gender-api.com

5 Gender definition, see https://whc.unesco.org/en/glossary/249

6
https://www.maxqda.com

7
https://sprweb.org/diversity-and-inclusivity-in-spr/

8
https://www.nature.com/nature-portfolio/about/diversity-commitment

9
https://www.unesco.org/reports/science/2021/en
