# Supplementary material for: Exploring individual biases in BCI research and users: Does gender matter?
Source: Front Hum Neurosci. 2026 Apr 17;19:1695370. doi: 10.3389/fnhum.2025.1695370 (PMC13133739; doi:10.3389/fnhum.2025.1695370)
Supplement: Supplementary file 1 [file Table_1.pdf]

Exploring Individual Biases in BCI Research and Users: Does Gender Matter?

Short Title: Gender Dynamics in BCI

Cornelia Herbert[1,*], **Viviana Ramos Acuna**[1], **Raphael R. K. Kneipp**[1], **Nina I. Kapfer**[1]

[1]*Applied Emotion and Motivation Psychology, Ulm University, Germany*

**Correspondence: cornelia.herbert@uni-ulm.de*

Correspondence*:
Cornelia Herbert

Applied Emotion and Motivation Psychology, Ulm University, Albert-Einstein-Allee 47, 89081 Ulm, Germany,
cornelia.herbert@uni-ulm.de

Supplement

Table 11b. Comprehensive summary of all eligible studies from the literature search in the publication period from 1980 to 2025. Hits include the following article types: reviews (R), research articles (A), and multisite studies combining analyses across datasets (D). Hits

from the specific search appear first (see the upper lines of Table 11 [2-3]; for details, see the text). Hits from the broad search (*N* = 51) follow next. 'Not specified' means that the critical information was not included in the title or abstract; for an explanation, see the Methods and Results sections. The reference for each publication is listed in the first column of the table, titled Ref. The references are numbered, and the complete reference list for the studies in Table 11b is provided in the supplementary material.

Ref	Type	Year	Title	Participants (N)	Number women (N)	Number men (N)	Healthy Participants vs. Patient Groups	Age of Participants	Method	Abstract Summary	Gender- or sex-differences	Gender- or sex-differences observed	Results
					Specific Search: EEG & BCI & gender/sex differences								
1	D	2024	Large scale investigation of the effect of gender on mu rhythm suppression in motor imagery brain-computer interfaces	248	124	124	Healthy individuals	Not specified	EEG	Investigation of the influence of gender on mu rhythm suppression in MI-BCIs.	Yes: gender	No	Gender was no significant predictor of BCI performance.
2	D	2010	BCI demographics: how many (and what kinds of) people can use an SSVEP BCI?	86	Not specified	Not specified	Healthy individuals	Not specified	EEG	Investigation of BCI demographics by analyzing the relationships between BCI performance, personal preferences, and participant factors like age and gender.	Yes: gender	Yes	Performance was better among younger participants and females.
3	D	2011	BCI demographics II: how many (and what kinds of) people can use a high-frequency SSVEP BCI?	86	Not specified	Not specified	Healthy individuals	Not specified	EEG	Investigation of BCI performance with high-frequency SSVEPs with medium and high frequencies as a function of demographic factors.	Gender or sex not reported	No	No significant impact of demographic variables, including age and gender, on BCI performance.
	Broad Search: EEG & gender/sex differences (in numerical/alphabetic order of authors, see reference list in the supplement)												
4	R	2001	Sleep EEG, depression and gender	Not specified	Not specified	Not specified	Not specified	Not specified	EEG	A review exploring the link between sleep, major depressive disorders (MDD), and gender, highlighting the lack of research on gender differences in sleep.	Yes: gender	Yes	Women are more vulnerable to depression because of gender differences in brain organization.

frontiers

5	A	2016	EEG alpha asymmetry as a gender-specific predictor of outcome to acute treatment with different antidepressant medications in the randomized iSPOT-D study	1344	336	1008	Patients with MDD and healthy controls	Not specified	EEG	Investigation of whether EEG alpha asymmetry distinguishes MDD outpatients from controls and predicts treatment outcomes.	Yes: gender	Yes	No difference in EEG alpha between groups; however, a significant gender effect for frontal alpha asymmetry. Greater right frontal alpha in women was associated with favorable responses to certain SSRIs.
6	A	2017	Gender Differences in Spontaneous and Evoked Activities of the Human Brain	21	11	10	Healthy individuals	18-27 years	EEG	Investigation of sex-related differences in spontaneous and evoked brain activity.	Yes: gender	Yes and no	No gender differences in stimulus recognition efficiency. Men showed lower EEG amplitudes in the resting state.
7	CP	2019	Investigating Sex Differences in Classification of Five Emotions from EEG and Eye Movement Signals	30	15	15	Healthy individuals	Not specified	EEG	Investigation of sex differences in classifying emotions from EEG and eye movement signals.	Yes: sex	Yes	Higher accuracy with same-sex strategies and significant differences in brain activity between genders.
8	A	2018	Sex Differences in Electrophysiology: P200 Event-related Potential Evidence	30	15	15	Healthy individuals	39.0 years	EEG	EEG-ERP study examining P200 modulation in male and female participants.	Yes: sex	Yes	Men had larger P200 amplitudes and shorter latencies compared to women.
9	A	2016	Pay attention to me! Late ERPs reveal gender differences in attention allocated to romantic partners	Not specified	Not specified	Not specified	Healthy individuals	Not specified	EEG	Investigation of gender differences in neural responses to romantic partners.	Yes: gender	Yes	Relationship quality factors influenced attention differently in women and men.
10	A	2016	Gender Differences in Quantitative Electroencephalogram During a Simple Hand Movement Task in Young Adults	36	18	18	Healthy individuals	Not specified	EEG	Investigation of gender differences in the EEG during hand movements.	Yes: gender	Yes	Greater power decreases were observed for women in all analyzed frequency bands.
11	A	2001	The effects of age and gender on sleep EEG power spectral density in the middle years of life (ages 20-60 years old)	100	Not specified	Not specified	Healthy individuals	20-60 years	EEG	Investigation of the effects of age and gender on sleep EEG power spectral density.	Yes: gender	Yes	Gender effects varied by frequency; women showed higher power density in delta, theta, and alpha bands.
12	A	2008	Gender Differences in the Mu Rhythm of the Human Mirror-Neuron System	40	20	20	Healthy individuals	Not specified	EEG	Investigation of mu rhythm activity in males and females during observation of hand actions.	Yes: gender	Yes	Women showed significantly stronger mu suppression than men during hand-action observation.

13	A	1993	Gender Differences in the EEG During Cognitive Activity	16	8	8	Healthy individuals	Adults	EEG	Investigation of EEG activity during cognitive tasks.	Yes: gender	Yes	Men had higher beta power; women had higher alpha power during cognitive tasks.
14	A	1989	Sex Differences in the Sleep EEG of Young Adults	28	15	13	Healthy individuals	21.9 years (mean)	EEG	Investigation of sleep EEG.	Yes: sex	Yes	Women had higher EEG power density throughout the sleep episode than men.
15	A	2019	EEG spectral powers and source localization in depressing, sad, and fun music videos focusing on gender differences	30	15	15	Healthy individuals	Not specified	EEG	Investigation of EEG power spectra and sources between genders while watching emotional music videos.	Yes: gender	Yes	Significant differences in EEG power and spatial distribution between women and men. Women exhibited higher EEG power across all frequency bands; different brain networks were recruited in response to emotional stimuli.
16	A	2023	Sex-related patterns in the electroencephalogram and their relevance in machine learning classifiers	1140	Not specified	Not specified	Patients with neurological disorders	Not specified	EEG	Investigation of sex differences in EEG data used for machine learning classifiers.	Yes: sex	Yes	Sex-related patterns could influence disease detection.
17	A	1995	Sleep EEG changes in psychotic disorders: gender and age effects	61	23	38	Patients with psychotic disorders	Not specified	EEG	Investigation of gender and age effects on sleep EEG in psychotic patients.	Yes: gender	Yes	Older men had less slow-wave sleep than women. Women showed a decline in REM sleep with age.
18	A	2000	Gender differences in the EEG of abstinent cocaine abusers	40	20	20	Patients with substance use disorders	21-41 years	EEG	Investigation of abstinent cocaine-abusing people.	Yes: gender	Yes	Cocaine-abusing women showed more normal EEG patterns compared to men.
19	A	2011	Sex differences in human EEG theta oscillations during spatial navigation in virtual reality	27	14	13	Healthy individuals	Young adults	EEG	Investigation of theta oscillations during spatial navigation.	Yes: sex	Yes	Women showed increased theta oscillations, with higher theta power correlating with better navigation performance.
20	A	2024	Preliminary Study on Gender Differences in EEG-Based Emotional Responses in Virtual Architectural Environments	20	10	10	Healthy individuals	Not specified	EEG	Investigation of gender differences in emotional responses to virtual environments.	Yes: gender	Yes	Females experienced more discomfort in virtual environments, while males showed more positive emotional responses.

21	A	2024	Effects of gender and age on sleep EEG functional connectivity differences in subjects with mild difficulty falling asleep	78	Not specified	Not specified	Individuals with sleep difficulties	Not specified	EEG	Investigation of how age and gender affect sleep EEG functional connectivity.	Yes: gender	Yes	Women had higher functional connectivity in high-frequency bands. There were significant differences in connectivity strength and sleep staging accuracy based on gender and age.
22	CP	2024	Exploring Individual and Sex Differences from Brain EEG Signals under Music Stimuli	Not specified	Not specified	Not specified	Not specified	Not specified	EEG	Investigation of EEG responses to music stimuli.	Yes: sex	Yes	Significant individual and gender-related differences in brain activity patterns.
23	A	2011	EEG power spectra response to a 4-h phase advance and gaboxadol treatment in 822 men and women	822	Not specified	Not specified	Healthy adults	18-64 years	EEG	Investigation of medication on EEG in transient insomnia.	Yes: sex	Yes	Women showed increased low-frequency activity and a greater treatment response than men.
24	R	2018	Gender effects on auditory P300: A systematic review	Not specified	Not specified	Not specified	Not specified	Not specified	EEG	A review on gender differences in P300 potentials.	Yes: gender	Yes	P300 amplitude was higher in women; minimal gender effects on P300 latency.
25	CP	2019	Sex differences observed in a study of EEG of linguistic activity and resting-state	16	8	8	Healthy individuals	Adults	EEG	Investigation of EEG signals during linguistic tasks and resting states.	Yes: sex	Yes	Males showed stronger theta and gamma activity in the right hemisphere during resting states.
26	A	2003	State markers of depression in sleep EEG: dependency on drug and gender in patients treated with tianeptine or paroxetine	38	Not specified	Not specifi ed	Patients with depression	Not specified	EEG	Investigation of medication on sleep EEG in depressed patients.	Yes: gender	Yes	Gender differences in treatment response and EEG sleep markers.
27	A	2024	A gender recognition method based on EEG microstates	216	74 (first dataset)	74 (first datas et)	Healthy individuals	Not specified	EEG	Investigation of EEG microstate parameters for gender classification.	Yes: gender	Yes	High classification accuracy, significant differences in microstate dynamics between women and men. Women showed higher temporal parameters and microstate complexity than men.

28	A	1999	Gender differences in late positive components evoked by human faces	Not specified	Not specified	Not specified	Healthy individuals	Not specified	EEG	Investigation of event-related potentials (ERPs) in response to faces.	Yes: gender	Yes	Larger P300 amplitudes in men and different response patterns in women based on face gender.
29	A	2023	Identifying sex differences in EEG-based emotion recognition using graph convolutional network with attention mechanism	Multiple datasets	Not specified	Not specified	Healthy individuals	Not specified	EEG	Investigation of sex differences in emotional EEG patterns across various datasets.	Yes: sex	Yes	Results revealed different key features in women and men.
30	A	2011	Gender differences in EEG coherent activity before and after training navigation skills in virtual environments	45	22	23	Healthy individuals	Young adults	EEG	Investigation of EEG changes during navigation tasks before and after navigation training.	Yes: gender	Yes	Gender differences in theta-band coherence and connectivity patterns: women showed higher coherence in the theta band before training; training altered connectivity patterns differently by gender.
31	R	2022	Sex differences in cognitive processing: An integrative review of electrophysiological findings	Not specified	Not specified	Not specified	Not specified	Not specified	EEG	A review on sex differences in brain electrical activity during cognitive processing.	Yes: sex	Yes	Consistent differences between men and women in visuospatial reasoning and language. Further research is needed.
32	A	2004	Gender differences in hemispheric organization during divergent thinking: an EEG investigation in human subjects	63	27	36	Healthy individuals	Not specified	EEG	Investigation of EEG patterns during divergent thinking.	Yes: gender	Yes	Gender-related differences in EEG coherence and amplitude suggest different cognitive strategies between men and women.
33	A	2003	Alcoholism risk and the P300 event-related brain potential: modality, task, and gender effects	48	24	24	Healthy young adults	Not specified	EEG	Investigation of P300 ERP modulation in young adults at low and high risk for alcoholism.	Yes: gender	Yes	Gender and task effects on P300 amplitudes.
			Behavioral and physiological findings of							Investigation of gender differences in global-			Women had lateralized P100 responses and greater P300

34	A	2006	gender differences in global-local visual processing	30	15	15	Healthy adults	Not specified	EEG	local visual processing using ERPs.	Yes: gender	Yes	responses for local stimuli compared to men.
35	A	2025	Impact of sex differences on subject-independent EEG-based emotion recognition models	Not specified	Not specified	Not specified	Not specified	Not specified	EEG	Investigation of how sex differences affect EEG-based emotion recognition.	Yes: sex	Yes	Women showed greater activation in the left hemisphere, while males showed greater activation in the right hemisphere during emotional tasks, including information about participants' sex improved model accuracy.
36	A	2010	Sex and individual differences in induced and evoked EEG measures of action observation	Not specified	Not specified	Not specified	Not specified	Not specified	EEG	Investigation of EEG responses to action observation.	Yes: sex	Yes	Differences in mu power and readiness potentials indicating different mirroring processes in women and men.
37	A	1998	P300 subcomponent abnormalities in schizophrenia: I. Physiological evidence for gender and subtype specific differences in regional pathology	113	23	42	Patients with schizophrenia	Not specified	EEG	Investigation of P300 subcomponents in schizophrenia.	Yes: gender	Yes	Women had greater decreases in specific P300 subcomponents.
38	A	2022	Sex differences of brain oscillatory activity and quantitative sensory testing in people with chronic osteoarthritis pain	108	90	18	Patients with chronic pain	69 years (median)	EEG	Investigation of sex differences in EEG and sensory testing in chronic pain patients.	Yes: sex	Yes	Women showed less oscillatory activity and lower pain thresholds compared to men.
39	A	2009	Gender differences in the mu rhythm during empathy for pain: An electroencephalographic study	32	16	16	Healthy individuals	Not specified	EEG	Investigation of mu rhythm suppression during pain empathy.	Yes: gender	Yes	Women showed stronger mu suppression than males in response to painful stimuli.
40	A	2024	Age-Related Aspects of Sex Differences in Event-Related Brain	155	Not specified	Not specified	Healthy individuals	18-65 years	EEG	Investigation of sex differences in event-related oscillations	Yes: sex	Yes	Women showed higher theta power in younger age groups, which declined with age. Men had more stable theta power.

			Oscillatory Responses: A Turkish Study							across different age groups.			
41	A	2021	Sex differences in resting EEG in healthy young adults	80	40	40	Healthy individuals	20.4 years (18-26 years)	EEG	Investigation of differences in resting EEG patterns in women and men.	Yes: sex	Yes	Females showed greater amplitudes across multiple frequency bands than males.
42	A	2007	Brain oscillations are highly influenced by gender differences	32	16	16	Healthy individuals	Not specified	EEG	Investigation of gender differences in event-related oscillations during visual stimulation.	Yes: gender	Yes	Gender influences brain oscillations.
43	R	2019	Cognitive sex differences and hemispheric asymmetry: A critical review of 40 years of research	Not specified	Not specified	Not specified	Not specified	Not specified	EEG	A review of the relationship between cognitive sex differences and hemispheric asymmetry.	Yes: sex	Yes and no	Stronger hemispheric asymmetry in men than in women, but the difference did not improve spatial or decrease verbal performance.
44	A	1999	P300, handedness, and corpus callosal size: gender, modality, and task	80	40	40	Healthy individuals	Young adults	EEG	Investigation of P300 ERPs in relation to handedness and gender.	Yes: gender	Yes	Larger P300 amplitudes in left-handed subjects and smaller P300 amplitudes in men compared to women.
45	A	2004	Gender differences in the cortical electrophysiological processing of visual emotional stimuli	30	15	15	Healthy individuals	Not specified	EEG	Investigation of gender differences in the processing of emotional visual stimuli.	Yes: gender	Yes	Women showed greater reductions in ERP latency for unpleasant images than men.
46	A	2013	Gender difference in event-related potentials to masked emotional stimuli in the oddball task	24	12	12	Healthy individuals	Not specified	EEG	Investigation of ERP responses to subliminally presented threat-related stimuli.	Yes: gender	Yes	Only women showed significant increases in the N170 and EPN EEG-ERP components.
47	A	2010	Gender differences in implicit and explicit				Not specified	Not specified	EEG		Yes: gender	Yes	Men showed earlier theta synchronization; women showed

			processing of emotional facial expressions as revealed by event-related theta synchronization	Not specified	Not specified	Not specified				Investigation of gender differences in emotional processing.			greater synchronization during later processing stages.
48	A	2015	Gender effect in human brain responses to bottom-up and top-down attention using the EEG 3D-Vector Field Tomography	20	10	10	Healthy individuals	32 (mean age)	EEG	Investigation of gender differences in attention allocation.	Yes: gender	Yes	Women had higher P300 amplitudes. The activation was located in different brain regions compared to males.
49	R	1984	Electrophysiological Correlates of Gender Differences: A Review	Not specified	Not specified	Not specified	Not specified	Not specified	EEG	A review of the electrophysiological correlates of gender differences.	gender	Yes	Overview of gender differences in electrophysiology.
50	A	2008	Gender-selective effects of the P300 and N400 components of the visual evoked potential	30	15	15	Healthy individuals	Not specified	EEG	An investigation of gender effects on VEP components.	Yes: gender	Yes	Women had greater P300 and N400 responses compared to men, with no significant differences in latency for any ERP component.
51	A	2018	From swing to cane: Sex differences of EEG resting-state temporal patterns during maturation and aging	179	90	89	Healthy individuals	6-87 years	EEG	Investigation of age and sex-related changes in EEG microstates.	Yes: sex	Yes	Men and women differed in microstate duration and occurrence; implications for neuropsychiatric diseases.
52	A	2015	Brain source localization of MMN, P300 and N400: Aging and gender differences	45	Not specified	Not specified	Healthy individuals	Young and elderly	EEG	Investigation of neuronal generators during an ERP study.	Yes: gender	Yes	Significant age and gender-related differences in MMN and P300 amplitudes.
53	A	2004	Gender differences in event-related potentials during visual-spatial attention	20	10	10	Healthy individuals	18-24 years	EEG	Investigation of gender differences in ERPs during a visual-spatial attention task.	Yes: gender	Yes	Men had higher amplitudes in P1 and P3b, whereas women had higher amplitudes in N1 than men.

frontiers

54	A	2020	Differences in Auditory P300 Event-Related<br><br>Potential in the Indian Population	34	21	13	Healthy individuals	20-25 years		Investigation of the P300 amplitude and latency measures in healthy control participants taken from research on schizophrenia	Yes: gender	Trend	Trend towards longer P300 latency in women compared to men. No difference in P300 amplitude.

Reference list of Table 11b in the order as they appear chronologically in Table 11b, as indicated by the numbers in the column references.

[1] Von Groll, V. G., Leeuwis, N., Rimbert, S., Roc, A., Pillette, L., Lotte, F., et al. (2024). Large scale investigation of the effect of gender on mu rhythm suppression in motor imagery brain-computer interfaces. *Brain-Computer Interfaces* 11, 87–97. doi: 10.1080/2326263X.2024.2345449

[2] Allison, B., Luth, T., Valbuena, D., Teymourian, A., Volosyak, I., and Graser, A. (2010). BCI demographics: how many (and what kinds of) people can use an SSVEP BCI? *IEEE Trans Neural Syst Rehabil Eng* 18, 107–116. doi: 10.1109/TNSRE.2009.2039495

[3] Volosyak, I., Valbuena, D., Lüth, T., Malechka, T., and Gräser, A. (2011). BCI demographics II: how many (and what kinds of) people can use a high-frequency SSVEP BCI? *IEEE Trans Neural Syst Rehabil Eng* 19, 232–239. doi: 10.1109/TNSRE.2011.2121919

[4] Armitage, R., and Hoffmann, R. F. (2001). Sleep EEG, depression and gender. *Sleep Med Rev* 5, 237–246. doi: 10.1053/smrv.2000.0144

[5] Arns, M., Bruder, G., Hegerl, U., Spooner, C., Palmer, D. M., Etkin, A., et al. (2016). EEG alpha asymmetry as a gender-specific predictor of outcome to acute treatment with different antidepressant medications in the randomized iSPOT-D study. *Clin Neurophysiol* 127, 509–519. doi: 10.1016/j.clinph.2015.05.032

[6] Aslanyan, E. V., Kiroy, V. N., Bakhtin, O. M., Minyaeva, N. R., Lazurenko, D. M., and Tambiev, A. E. (2017). Gender Differences in Spontaneous and Evoked Activities of the Human Brain. *Hum Physiol* 43, 644–652. doi: 10.1134/S0362119717040041

[7] Bao, L.-Q., Qiu, J.-L., Tang, H., Zheng, W.-L., and Lu, B.-L. (2019). Investigating Sex Differences in Classification of Five Emotions from EEG and Eye Movement Signals., in *2019 41st Annual International Conference of the IEEE Engineering in Medicine and Biology Society (EMBC)*, (Berlin, Germany: IEEE), 6746–6749. doi: 10.1109/EMBC.2019.8857476

[8] Bourisly, A. K., and Shuaib, A. (2018). Sex Differences in Electrophysiology: P200 Event-related Potential Evidence. *Transl Neurosci* 9, 72–77. doi: 10.1515/tnsci-2018-0013

[9] Burdwood, E. N., and Simons, R. F. (2016). Pay attention to me! Late ERPs reveal gender differences in attention allocated to romantic partners. *Psychophysiology* 53, 436–443. doi: 10.1111/psyp.12589

[10] Cantillo-Negrete, J., Carino-Escobar, R. I., Carrillo-Mora, P., Flores-Rodríguez, T. B., Elías-Vinas, D., and Gutiérrez-Martínez, J. (2016). Gender Differences in Quantitative Electroencephalogram During a Simple Hand Movement Task in Young Adults. *Rev Invest Clin* 68, 245–255.

[11] Carrier, J., Land, S., Buysse, D. J., Kupfer, D. J., and Monk, T. H. (2001). The effects of age and gender on sleep EEG power spectral density in the middle years of life (ages 20-60 years old). *Psychophysiology* 38, 232–242.

[12] Cheng, Y., Lee, P.-L., Yang, C.-Y., Lin, C.-P., Hung, D., and Decety, J. (2008). Gender Differences in the Mu Rhythm of the Human Mirror-Neuron System. *PLoS ONE* 3, e2113. doi: 10.1371/journal.pone.0002113

[13] Corsi-Cabrera, M., Ramos, J., Guevara, M. A., Arce, C., and Gutierrez, S. (1993). Gender Differencesm in the Eeg During Cognitive Activity. *International Journal of Neuroscience* 72, 257–264. doi: 10.3109/00207459309024114

[14] Dijk, D. J., Beersma, D. G. M., and Bloem, G. M. (1989). Sex Differences in the Sleep EEG of Young Adults: Visual Scoring and Spectral Analysis. *Sleep* 12, 500–507. doi: 10.1093/sleep/12.6.500

[15] Goshvarpour, A., and Goshvarpour, A. (2019). EEG spectral powers and source localization in depressing, sad, and fun music videos focusing on gender differences. *Cogn Neurodyn* 13, 161–173. doi: 10.1007/s11571-018-9516-y

[16] Jochmann, T., Seibel, M. S., Jochmann, E., Khan, S., Hämäläinen, M. S., and Haueisen, J. (2023). Sex-related patterns in the electroencephalogram and their relevance in machine learning classifiers. *Human Brain Mapping* 44, 4848–4858. doi: 10.1002/hbm.26417

[17] Keshavan, M. S., Reynolds, C. F., Haas, G., Miewald, J. M., and Montrose, D. M. (1995). Sleep EEG changes in psychotic disorders: gender and age effects. *Neuropsychobiology* 32, 1–8. doi: 10.1159/000119204

[18] King, D. E., Herning, R. I., Gorelick, D. A., and Cadet, J. L. (2000). Gender differences in the EEG of abstinent cocaine abusers. *Neuropsychobiology* 42, 93–98. doi: 10.1159/000026678

frontiers

[19] Kober, S. E., and Neuper, C. (2011). Sex differences in human EEG theta oscillations during spatial navigation in virtual reality. *International Journal of Psychophysiology* 79, 347–355. doi: 10.1016/j.ijpsycho.2010.12.002

[20] Li, Z., Wang, K., Hai, M., Cai, P., and Zhang, Y. (2024). Preliminary Study on Gender Differences in EEG-Based Emotional Responses in Virtual Architectural Environments. *Buildings* 14, 2884. doi: 10.3390/buildings14092884

[21] Luo, X., Zhou, B., Shi, J., Li, G., and Zhu, Y. (2024a). Effects of gender and age on sleep EEG functional connectivity differences in subjects with mild difficulty falling asleep. *Front Psychiatry* 15, 1433316. doi: 10.3389/fpsyt.2024.1433316

[22] Luo, Y., Nishida, S., and Kobayashi, I. (2024b). Exploring Individual and Sex Differences from Brain EEG Signals under Music Stimuli., in *2024 Joint 13th International Conference on Soft Computing and Intelligent Systems and 25th International Symposium on Advanced Intelligent Systems (SCIS&ISIS)*, (Himeji, Japan: IEEE), 1–4. doi: 10.1109/SCISISIS61014.2024.10760242

[23] Ma, J., Dijk, D.-J., Svetnik, V., Tymofyeyev, Y., Ray, S., Walsh, J. K., et al. (2011). EEG power spectra response to a 4-h phase advance and gaboxadol treatment in 822 men and women. *J Clin Sleep Med* 7, 493-501A. doi: 10.5664/JCSM.1316

[24] Melynyte, S., Wang, G. Y., and Griskova-Bulanova, I. (2018). Gender effects on auditory P300: A systematic review. *International Journal of Psychophysiology* 133, 55–65. doi: 10.1016/j.ijpsycho.2018.08.009

[25] Moctezuma, L. A., and Molinas, M. (2019). Sex differences observed in a study of EEG of linguistic activity and resting-state: Exploring optimal EEG channel configurations., in *2019 7th International Winter Conference on Brain-Computer Interface (BCI)*, (Gangwon, Korea (South): IEEE), 1–6. doi: 10.1109/IWW-BCI.2019.8737312

[26] Murck, H., Nickel, T., Künzel, H., Antonijevic, I. A., Schill, J., Zobel, A., et al. (2003). State markers of depression in sleep EEG: dependency on drug and gender in patients treated with tianeptine or paroxetine. *Neuropsychopharmacology* 28, 348–358. doi: 10.1038/sj.npp.1300029

[27] Niu, Y., Chen, X., Chen, Y., Yao, Z., Chen, X., Liu, Z., et al. (2024). A gender recognition method based on EEG microstates. *Computers in Biology and Medicine* 173, 108366. doi: 10.1016/j.compbiomed.2024.108366

[28] Oliver-Rodríguez, J. C., Guan, Z., and Johnston, V. S. (1999). Gender differences in late positive components evoked by human faces. *Psychophysiology* 36, 176–185.

[29] Peng, D., Zheng, W.-L., Liu, L., Jiang, W.-B., Li, Z., Lu, Y., et al. (2023). Identifying sex differences in EEG-based emotion recognition using graph convolutional network with attention mechanism. *J. Neural Eng.* 20, 066010. doi: 10.1088/1741-2552/ad085a

[30] Ramos-Loyo, J., and Sanchez-Loyo, L. M. (2011). Gender differences in EEG coherent activity before and after training navigation skills in virtual environments. *Fiziol Cheloveka* 37, 68–75.

[31] Ramos-Loyo, J., González-Garrido, A. A., Llamas-Alonso, L. A., and Sequeira, H. (2022). Sex differences in cognitive processing: An integrative review of electrophysiological findings. *Biological Psychology* 172, 108370. doi: 10.1016/j.biopsycho.2022.108370

[32] Razumnikova, O. M. (2004). Gender differences in hemispheric organization during divergent thinking: an EEG investigation in human subjects. *Neuroscience Letters* 362, 193–195. doi: 10.1016/j.neulet.2004.02.066

[33] Reese, C., and Polich, J. (2003). Alcoholism risk and the P300 event-related brain potential: modality, task, and gender effects. *Brain Cogn* 53, 46–57. doi: 10.1016/s0278-2626(03)00202-1

[34] Roalf, D., Lowery, N., and Turetsky, B. I. (2006). Behavioral and physiological findings of gender differences in global-local visual processing. *Brain Cogn* 60, 32–42. doi: 10.1016/j.bandc.2005.09.008

[35] Sheoran, A., and Valderrama, C. E. (2025). Impact of sex differences on subject-independent EEG-based emotion recognition models. *Computers in Biology and Medicine* 190, 110036. doi: 10.1016/j.compbiomed.2025.110036

[36] Silas, J., Levy, J. P., Nielsen, M. K., Slade, L., and Holmes, A. (2010). Sex and individual differences in induced and evoked EEG measures of action observation. *Neuropsychologia* 48, 2417–2426. doi: 10.1016/j.neuropsychologia.2010.03.004

[37] Turetsky, B. I., Colbath, E. A., and Gur, R. E. (1998). P300 subcomponent abnormalities in schizophrenia: I. Physiological evidence for gender and subtype specific differences in regional pathology. *Biol Psychiatry* 43, 84–96. doi: 10.1016/S0006-3223(97)00258-8

[38] Vasquez-Avila, K., Pacheco-Barrios, K., Simis, M., Imamura, M., Battistella, L., and Fregni, F. (2022). Sex differences of brain oscillatory activity and quantitative sensory testing in people with chronic osteoarthritis pain: a cross-sectional study. *The Lancet Rheumatology* 4, S18. doi: 10.1016/S2665-9913(22)00294-6

[39] Yang, C.-Y., Decety, J., Lee, S., Chen, C., and Cheng, Y. (2009). Gender differences in the mu rhythm during empathy for pain: An electroencephalographic study. *Brain Research* 1251, 176–184. doi: 10.1016/j.brainres.2008.11.062

[40] Yener, G., Kıyı, İ., Düzenli-Öztürk, S., and Yerlikaya, D. (2024). Age-Related Aspects of Sex Differences in Event-Related Brain Oscillatory Responses: A Turkish Study. *Brain Sciences* 14, 567. doi: 10.3390/brainsci14060567

[41] Cave, A. E., and Barry, R. J. (2021). Sex differences in resting EEG in healthy young adults. *International Journal of Psychophysiology* 161, 35–43. doi: 10.1016/j.ijpsycho.2021.01.008

[42] Güntekin, B., and Başar, E. (2007). Brain oscillations are highly influenced by gender differences. *International Journal of Psychophysiology* 65, 294–299. doi: 10.1016/j.ijpsycho.2007.03.009

[43] Hirnstein, M., Hugdahl, K., and Hausmann, M. (2019). Cognitive sex differences and hemispheric asymmetry: A critical review of 40 years of research. *Laterality: Asymmetries of Body, Brain and Cognition* 24, 204–252. doi: 10.1080/1357650X.2018.1497044

[44] Hoffman, L. D., and Polich, J. (1999). P300, handedness, and corpus callosal size: gender, modality, and task. *International Journal of Psychophysiology* 31, 163–174. doi: 10.1016/S0167-8760(98)00050-6

[45] Kemp, A. H., Silberstein, R. B., Armstrong, S. M., and Nathan, P. J. (2004). Gender differences in the cortical electrophysiological processing of visual emotional stimuli. *NeuroImage* 21, 632–646. doi: 10.1016/j.neuroimage.2003.09.055

[46] Kim, E. Y., Lee, S.-H., Park, G., Kim, S., Kim, I., Chae, J.-H., et al. (2013). Gender difference in event related potentials to masked emotional stimuli in the oddball task. *Psychiatry Investig* 10, 164–172. doi: 10.4306/pi.2013.10.2.164

[47] Knyazev, G. G., Slobodskoj-Plusnin, J. Y., and Bocharov, A. V. (2010). Gender differences in implicit and explicit processing of emotional facial expressions as revealed by event-related theta synchronization. *Emotion* 10, 678–687. doi: 10.1037/a0019175

[48] Kosmidou, V. E., Adam, A., Papadaniil, C. D., Tsolaki, M., Hadjileontiadis, L. J., and Kompatsiaris, I. (2015). Gender effect in human brain responses to bottom-up and top-down attention using the EEG 3D-Vector Field Tomography. *Annu Int Conf IEEE Eng Med Biol Soc* 2015, 7574–7577. doi: 10.1109/EMBC.2015.7320145

[49] Shearer, D. E., Cohn, N. B., Dustman, R. E., and LaMarche, J. A. (1984). Electrophysiological Correlates of Gender Differences: A Review. *American Journal of EEG Technology* 24, 95–107. doi: 10.1080/00029238.1984.11080133

[50] Steffensen, S. C., Ohran, A. J., Shipp, D. N., Hales, K., Stobbs, S. H., and Fleming, D. E. (2008). Gender-selective effects of the P300 and N400 components of the visual evoked potential. *Vision Research* 48, 917–925. doi: 10.1016/j.visres.2008.01.005

[51] Tomescu, M. I., Rihs, T. A., Rochas, V., Hardmeier, M., Britz, J., Allali, G., et al. (2018). From swing to cane: Sex differences of EEG resting-state temporal patterns during maturation and aging. *Developmental Cognitive Neuroscience* 31, 58–66. doi: 10.1016/j.dcn.2018.04.011

[52] Tsolaki, A., Kosmidou, V., Hadjileontiadis, L., Kompatsiaris, I. (Yiannis), and Tsolaki, M. (2015). Brain source localization of MMN, P300 and N400: Aging and gender differences. *Brain Research* 1603, 32–49. doi: 10.1016/j.brainres.2014.10.004

[53] Vaquero, E., Cardoso, M. J., Vázque, M., and Gómez, C. M. (2004). GENDER DIFFERENCES IN EVENT-RELATED POTENTIALS DURING VISUAL-SPATIAL ATTENTION. *International Journal of Neuroscience* 114, 541–557. doi: 10.1080/00207450490422056

[54] Uvais, N. A., Nizamie, S. H., Das, B., Katshu, M. Z. U. H., and Sreeraj, V. S. (2020). Gender Differences in Auditory P300 Event-Related Potential in Indian Population. *Indian J Psychol Med* 42, 198–200. doi: 10.4103/IJPSYM.IJPSYM_329_19
